# Models that learn how humans learn: The case of decision-making and its disorders

**DOI:** 10.1371/journal.pcbi.1006903

**Published:** 2019-06-11

**Authors:** Amir Dezfouli, Kristi Griffiths, Fabio Ramos, Peter Dayan, Bernard W. Balleine

**Affiliations:** 1 School of Psychology, UNSW, Sydney, Australia; 2 Data61, CSIRO, Australia; 3 Westmead Institute for Medical Research, University of Sydney, Sydney, Australia; 4 University of Sydney, Sydney, Australia; 5 Gatsby Computational Neuroscience Unit, UCL, London, United Kingdom; 6 Max Planck Institute for Biological Cybernetics, Tübingen, Germany; Stiftung caesar, GERMANY

## Abstract

Popular computational models of decision-making make specific assumptions about learning processes that may cause them to underfit observed behaviours. Here we suggest an alternative method using recurrent neural networks (RNNs) to generate a flexible family of models that have sufficient capacity to represent the complex learning and decision- making strategies used by humans. In this approach, an RNN is trained to predict the next action that a subject will take in a decision-making task and, in this way, learns to imitate the processes underlying subjects’ choices and their learning abilities. We demonstrate the benefits of this approach using a new dataset drawn from patients with either unipolar (n = 34) or bipolar (n = 33) depression and matched healthy controls (n = 34) making decisions on a two-armed bandit task. The results indicate that this new approach is better than baseline reinforcement-learning methods in terms of overall performance and its capacity to predict subjects’ choices. We show that the model can be interpreted using off-policy simulations and thereby provides a novel clustering of subjects’ learning processes—something that often eludes traditional approaches to modelling and behavioural analysis.

## Author summary

Computational models of decision-making provide a quantitative characterisation of the learning and choice processes behind human actions. Designing a computational model is often based on manual engineering with an iterative process to examine the consistency between different aspects of the model and the empirical data. In practice, however, inconsistencies between the model and observed behaviours can remain hidden behind examined summary statistics. To address this limitation, we developed a recurrent neural network (RNNs) as a flexible type of model that can automatically characterize human decision-making processes without requiring tweaking and engineering. To show the benefits of this new approach, we collected data on a decision-making task conducted on subjects with either bipolar or unipolar depression, as well as healthy controls. The results showed that, indeed, important aspects of decision-making remained uncaptured by typical computational models and even their enhanced variants, but were captured by RNNs automatically. Further, we were able to show that the nature of such processes can be unveiled by simulating the model under various conditions. This new approach can be used, therefore, as a standalone model of decision-making or as a baseline model to evaluate how well other candidate models fit observed data.

## Introduction

A computational model of decision-making is a mathematical function that inputs past experiences—such as chosen actions and the value of rewards—and outputs predictions about future actions [e.g. 1, 2, 3]. Typically, experimenters develop such models by specifying a set of structural assumptions along with free parameters that allow the model to produce a range of behaviors. The models are then fitted to the observed behaviors in order to obtain the parameter settings that make the model’s predictions as close as possible to the empirical data. Nevertheless, if the actual learning and choice processes used by real human subjects differ from those assumptions, e.g., if a single learning-rate parameter is assumed to update the effects of reward and punishment on action values when they are in fact modulated by different learning-rates, then the model will misfit the data [e.g., 4]. To overcome this problem, computational modelling often involves an iterative process that includes additional analyses to assess assumptions about model behavior, subsequent emendation of the structural features of the model to reduce residual fitting error, then new analyses, and so forth. The final model is that which is simplest and misfits the least. This iterative process has been common practise for model development in domains such as cognitive science, computational psychiatry, and model-based analyses of neural data [e.g., 5, 6, 7, 8, 9, 10, 11]. This approach is, however, limited from two standpoints: (i) It is typically unclear when to stop iterating over models. This is because in each iteration the unexplained variance in the data can be either attributed to the natural randomness of humans actions, which implies that no further model improvement is required, or to the lack of a mechanism in the model to absorb the remaining variance, which implies that further iterations are required. (ii) Even if it is believed that further iterations are required, improving the model will be mostly based on manual engineering in the hope of finding a new mechanism that, when added to the model, provides a better explanation for the data.

Here to address these limitations we consider an alternative approach based on recurrent neural networks (RNNs); a flexible class of models that make minimal assumptions about the underlying learning processes used by the subject and that are known to have sufficient capacity to represent any form of computational process [12], including those believed to be behind the behaviour of humans and other animals in a wide range of decision-making, cognitive and motor tasks [13, 14, 15, 16, 17, 18, 19, 20, 21, 22, 23, 24]. Since these models are flexible, they can automatically characterize the major behavioral trends exhibited by real subjects without requiring tweaking and engineering. This is achieved by training a network to learn how humans learn [25, 26, 27, 28], which involves adjusting the weights in a network so that it can predict the choices that subjects make both during learning and at asymptote. At this point the weights are frozen and the model is simulated on the actual learning task to assess its predictive capacity and to gain insights into the subjects’ behavior. This approach is not prone to the problems mentioned earlier because RNNs can in principle be trained to represent any form of behavioural process without requiring manual engineering; however, a potential problem is that the models are so flexible that they may overfit the data and not generalize in a relevant manner; an issue that we address by using regularisation methods and cross-validation.

To illustrate and evaluate this approach, we focus on a relatively simple decision-making task, involving a two-arm bandit, in which subjects chose between two actions (button presses) that were rewarded probabilistically. To examine the predictive capacity of RNNs under typical and atypical conditions, data from three groups were collected: healthy subjects, and patients with either unipolar or bipolar depression. We found that RNNs were able to learn the subjects’ decision-making strategies more accurately than both baseline reinforcement-learning and logistic regression models. Furthermore, we show that off-policy simulations of the RNN model allowed us to visualize, and thus uncover, the properties of the learning process behind subjects’ actions and that these were inconsistent with the assumptions made by reinforcement-learning treatments. Furthermore, we illustrate how the RNN method can be applied to predict diagnostic categories for different patient populations.

## Results

### Model and task settings

#### RNN

**Fig 1:**
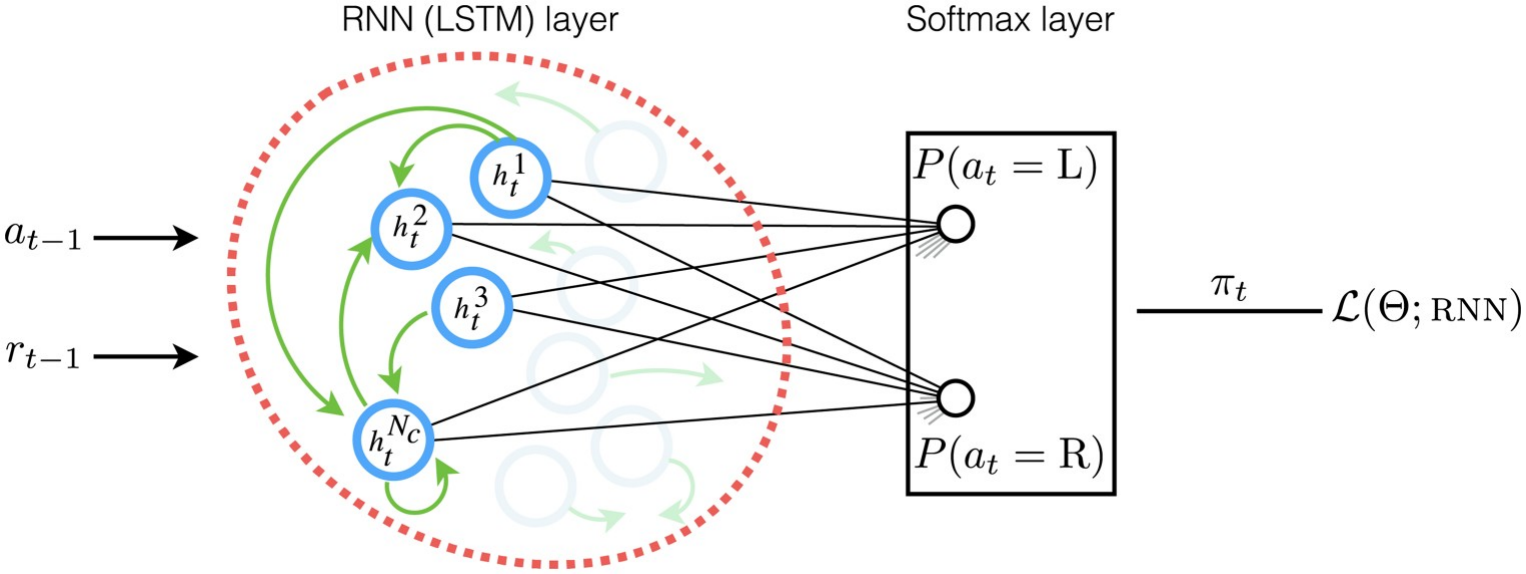
Structure of the rnn model. The model has an lstm layer (shown by red dashed line) which receives the previous action and reward as inputs, and is connected to a softmax layer (shown by a black rectangle) which outputs the probability of selecting each action on the next trial (policy). The lstm layer is composed of a set of lstm cells (*N*_*c*_ cells shown by blue circles), that are connected to each other (shown by green arrows). The outpt of the cells (denoted by $h_{t}^{i}$ for cell *i* at time *t*) are connected to a softmax layer using a set of connections shown by black lines. The free parameters of the model (in both lstm and softmax layers) are denoted by Θ, and L(Θ,rnn) is a metric which represents how well the model fits subjects’ data and is used to adjust the parameters of the model using the maximum-likelihood estimate as the network learns how humans learn.

The architecture we used is depicted in Fig 1; it is a particular form of recurrent neural network. The model is composed of an lstm layer [Long short-term memory; 29], which is a recurrent neural network, and an output softmax layer with two nodes (since there are two actions in the task). The inputs to the model on each trial are the previous action and the reward received after taking the action, and the outputs of the model are the probabilities of selecting each action on the next trial. We refer to the framework proposed here as rnn.

The lstm layer is composed of a set of interconnected lstm cells, in which each cell can be thought of as a memory unit which maintains and updates a scalar value over time (shown by $h_{t}^{i}$ in Fig 1 for the i*th* lstm cell at time *t*). On each trial, the value of each cell is updated based on the inputs and on the last value of the other lstm cells in the network (including the cell itself), and in this way the lstm layer can track relevant information regarding the history of past rewards and actions. Each lstm cell outputs its current value ($h_{t}^{i}$) to the softmax layer through an additional set of connections that determine the influence of the output of each cell on the predictions for the next action (shown by lines connecting them in Fig 1). As a whole, such an architecture is able to *learn* in a decision-making task by tracking the history of past experiences using the lstm layer, and then turning this information into subsequent actions through the outputs of the softmax layer.

The way in which a network learns in the task and maps past experiences to future actions is modulated by weights in the network. Here, our aim was to tune the weights so that the network could predict the next action taken by the subjects—given that the inputs to the network were the same as those that the subjects received on the task. This is learning how humans learn, in which the weights are trained to optimise a metric (denoted by L(Θ;rnn) in Fig 1) which represents how well the model predicts subjects’ choices. In order to prevent the model from overfitting the data, we used early stopping [a commonly used regularisation method in deep learning; 30] and used cross-validation to assess the generalization abilities of the model to predict unseen data.

#### Baseline models

We compared the predictive accuracy of the rnn model with classical exemplars from the reinforcement learning (RL) family as well as a logistic regression model. The first baseline RL model was the *Q*-learning model (denoted by ql), in which subjects’ choices are determined by learned action values [often called *Q* values; 31], which are updated based on the experience of reward [as used for example in 32]. The second baseline model was *Q*-learning with perseveration (denoted by qlp), which is similar to ql but has an extra parameter that allows for a tendency to stick with the same action for multiple trials (i.e., to perseverate), or sometimes to alternate between the actions [independently of reward effects; 4, 33]. As we show below, the accounts of subject choices provided by both ql and qlp were significantly worse than rnn, and so we developed a new baseline model that we called generalised *Q*-learning (denoted by gql). This model extends ql and qlp models by learning multiple values for each action using different learning rates, and also by tracking the history of past actions at different time scales. The final baseline model we used was a logistic regression model (denoted by lin) in which the probability of taking each action was determined by a linear combination of previous rewards, actions and their interactions [33, 34]. See section Computational models in Materials and methods for more details.

#### Task and subjects

**Fig 2:**
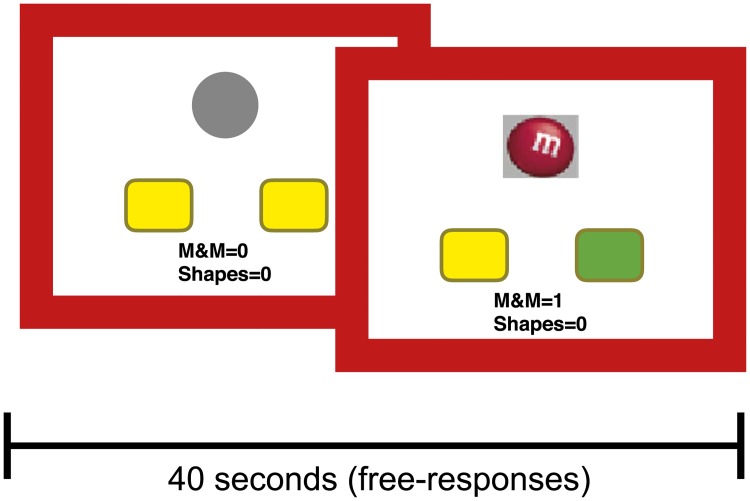
Structure of the decision-making task. Subjects had a choice between a left keypress (L) and a right keypress (R), shown by yellow rectangles. Before the choice, no indication was given as to which button was more likely to lead to reward. When the participant made a rewarded choice, the button chosen was highlighted (green) and a picture of the earned reward was presented for 500ms (M&M chocloate in this case). The task was divided into 12 different blocks each lasting for 40 seconds and separated by a 12-second inter-block interval. Within each block actions were self-paced and participants were free to complete as many trials as they could within the 40 second time limit. The probability of earning a reward from each action was varied between the blocks. See the text for more details about the probabilities of earning rewards from actions.

The instrumental learning task (Fig 2) involved participants choosing between pressing a left (L action) or right (R action) button (self-paced responses) in order to earn food rewards (an M&M chocolate or a BBQ flavoured cracker). The task was divided into 12 different blocks each lasting for 40 seconds and separated by a 12-second inter-block interval. Within each block one of the actions was better than the other in terms of the probability of earning a reward but across blocks the action with the higher reward probability was varied, i.e., in some of the blocks the left action was better while in others the right action was better. The reward probability for the better action was 0.25, 0.125, or 0.08 and the probability of earning reward from the other action was always 0.05 (probabilities were fixed within each block); as such, there were six pairs of reward probabilities and each was repeated twice. 34 uni-polar depression (depression), 33 bipolar (bipolar) and 34 control (healthy) participants (age, gender, IQ and education matched) completed the task. See Materials and methods for the details.

### Performance in the task

**Fig 3:**
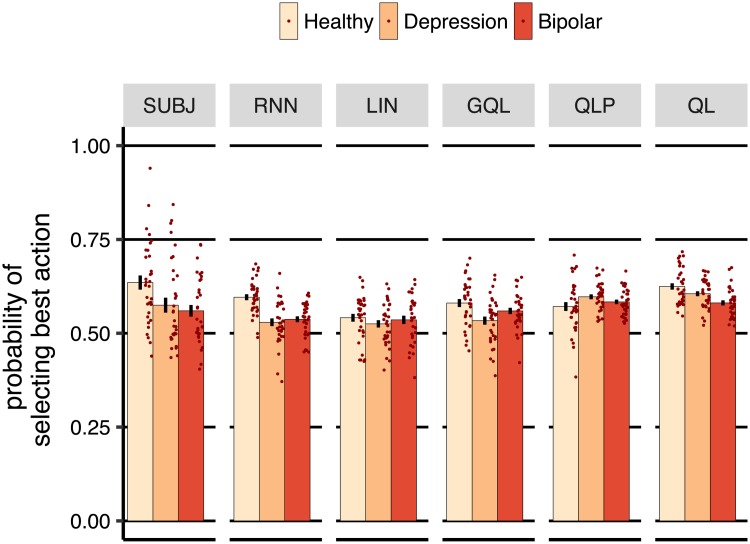
Probability of selecting the action with the higher reward probability (averaged over subjects). subj refers to the data of the experimental subjects, whereas the remaining columns show simulations of the models trained on the task (on-policy simulations) with the same reward probabilities and for the same number of trials that each subject completed. Each dot represents a subject and error-bars represent 1 SEM.

Fig 3 shows the probability of selecting the best action (i.e., the action with the higher reward probability). Results are shown by subject (i.e., subj) in the graph. The probability of selecting the better action was significantly higher than the other action in all groups (healthy [*η* = 0.270, SE = 0.026, *p* < 0.001], depression [*η* = 0.149, SE = 0.028, *p* < 0.001], bipolar [*η* = 0.119, SE = 0.021, *p* < 0.001]). Comparing healthy and depression groups revealed that the group x action interaction had a significant effect on the probability of selecting actions [*η* = −0.120, SE = 0.038, *p* = 0.002]. A similar effect was observed when comparing the healthy and bipolar groups [*η* = −0.150, SE = 0.034, *p* < 0.001]. In summary, these results indicate that all groups were able to direct their actions toward the better choice, however the depression and bipolar groups were less able to do so compared to the healthy group.

Next, we trained three instances of a rnn using the data from each group and then froze the weights of the models and simulated them on-policy in the task (with the same reward probabilities and for the same number of trials that each subject completed). On-policy means that the models completed the task on their own by selecting the actions that they predicted a representative subject would take in each situation. The results of the simulations are shown in Fig 3 in the rnn column. Similar to the subjects’ data, the probability of selecting the better action was significantly higher than the other action in all the three groups (healthy [*η* = 0.192, SE = 0.011, *p* < 0.001], depression [*η* = 0.058, SE = 0.014, *p* < 0.001], bipolar [*η* = 0.074, SE = 0.011, *p* < 0.001]). Therefore, although the structure of rnn was initially unaware that the objective of the task was to collect rewards, its actions were directed toward the better key by following the strategy that it learned from the subjects’ actions.

We also trained three instances of each baseline model using the data from each group, and simulated these on the task. Fig 3 shows the results of the simulations. As the graph shows, the lin model was also able to direct its choices toward the best action by learning the effect of past choices and rewards on the next actions. A similar pattern was observed for gql, qlp and ql models in the figure, which is not surprising as the structure of these models includes value representations which can be used for reward maximization. Thus, all of the models were consistent with the subjects’ performance in terms of being able to find and select the best actions in the task. Further details about the models’ parameters and training can be found in the Supporting information (estimated parameters for ql, qlp and gql models are shown in S3, S4 and S5 Tables respectively; the negative log-likelihood for each model is reported in S6 Table. See S8 Table for the effect of initialisation of the network on the negative log-likelihood of the trained rnn. See S7 Table for the negative log-likelihood when a separate model was fitted to each subject in the case of baseline models. See S3 Text for the analysis of randomness of choices).

### The immediate effect of reward on choice

**Fig 4:**
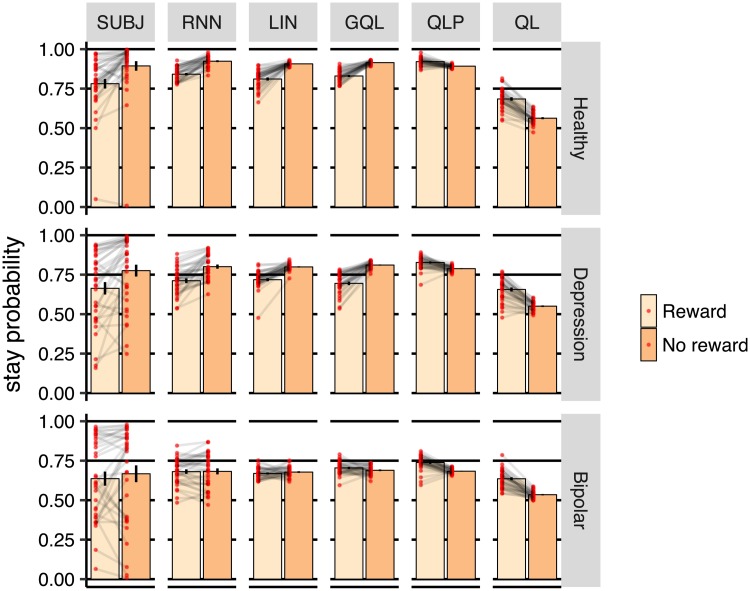
Probability of staying on the same action based on whether the previous trial was rewarded (reward) or not rewarded (no reward), averaged over subjects. subj shows the data from subjects and results of columns are derived from on-policy simulations of various models on the task. Each dot represents a subject and error-bars represent 1SEM.

The analyses in the previous section suggested that rnn was able to guide its actions toward the better choices, consistent with subjects’ behavior. However, there are multiple strategies that the models could follow to achieve this, and here we aimed to establish whether the strategy used by the models was similar to that used by the subjects’. We started by investigating the immediate effect of reward on choice. Fig 4 shows the effect of earning a reward on the previous trial on the probability of staying on the same action in the next trial. For the subjects (subj), earning a reward significantly *decreased* the probability of staying on the same action in the healthy and depression groups, but not in the bipolar group (healthy [*η* = 0.112, SE = 0.019, *p* < 0.001], depression [*η* = 0.111, SE = 0.029, *p* < 0.001], bipolar [*η* = 0.030, SE = 0.035, *p* = 0.391]). As the figure shows, the same pattern was observed in rnn (healthy [*η* = 0.082, SE = 0.006, *p* < 0.001], depression [*η* = 0.089, SE = 0.013, *p* < 0.001], bipolar [*η* = 0.001, SE = 0.010, *p* = 0.887]), which shows that the strategy used by rnn was similar to the subjects’ according to this analysis.

In contrast, stay probabilities were in the opposite directions in ql and qlp, i.e., the probability of staying on the same action was *higher* after earning reward (for the case of qlp; healthy [*η* = −0.028, SE = 0.004, *p* < 0.001], depression [*η* = −0.039, SE = 0.006, *p* < 0.001], bipolar [*η* = −0.054, SE = 0.007, *p* < 0.001]), which differs from the subjects’ data. This pattern was expected from baseline reinforcement-learning models, i.e., ql and qlp, because, in these models, earning reward increases the value of the taken action, which raises the probability of choosing that action on the next trial. Indeed, this learning process is embedded in the parametric forms of ql and qlp models, and cannot be reversed no matter what values are assigned to the free-parameters of these models. Therefore, although qlp and ql were able to find the best action in the task, analyzing the immediate effect of reward showed that their learning processes differed from those used by the subjects’.

To address this limitation of ql and qlp, we designed gql as a baseline model with more relaxed assumptions, in which action values could have an opposite effect on the probability of selecting actions, and so could generate a similar response pattern to the subjects’, as shown in the figure. The lin model was also able to produce a pattern similar to the empirical data. This is because in this model actions are determined by previous rewards (and actions) without making assumptions about the direction of the effects. Therefore, gql and lin appear to be able to model subjects’s choices, at least in terms of the behavioral summaries presented here and in the previous section. Despite this, it remains an open question whether these models can represent all of the behavioural trends in the data, or whether there are some missing trends that were undetected in the summary statistics. In the next section we will answer this question by comparing the prediction capacity of gql and lin with rnn, as a model that has the capacity to capture all of the behavioural trends in the data.

### Action prediction

Here our aim was to quantify how well the models predicted the actions chosen by the subjects. We used leave-one-out cross-validation for this purpose in which, at each round, one of the subjects was withheld and the model was trained using the remaining subjects; the trained model was then used to make predictions about the withheld subject. The withheld subject was rotated in each group, yielding 34, 34 and 33 prediction accuracy measures in the healthy, depression, and bipolar groups, respectively.

**Fig 5:**
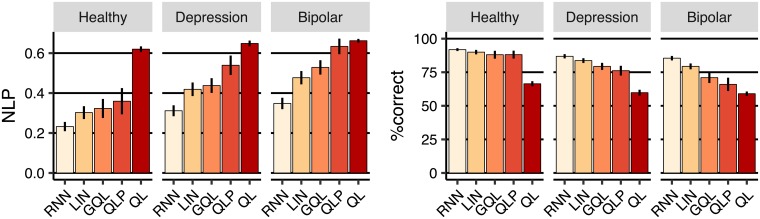
Cross-validation results. **(Left-panel)** nlp (negative log-probability) averaged across leave-one-out cross-validation folds. Lower values are better. **(Right-panel)** Percentage of actions predicted correctly averaged over cross-validation folds. Error-bars represent 1SEM.

The results are reported in Fig 5. The left-panel of the figure shows prediction accuracy in terms of nlp (negative log-probability; averaged over leave one-out cross-validation folds; lower values are better) and the right-panel shows the percentage of actions predicted correctly (‘%correct’; higher values are better). nlp roughly represents how well each model fits the choices of the withheld subject and so, unlike ‘%correct’, takes the certainty of predictions into account. Therefore, we focus on nlp in this analysis. Firstly, lin had the best nlp among the baseline models in all the three groups, although it was not statistically better than gql. The gql model was the second best among the baseline models and its advantage over qlp was statistically significant in the depression and bipolar groups (healthy [*η* = −0.036, SE = 0.020, *p* = 0.086], depression [*η* = −0.101, SE = 0.024, *p* < 0.001], bipolar [*η* = −0.105, SE = 0.019, *p* < 0.001]). Secondly, rnn’s nlp was even better than the best baseline model (lin) across all groups (healthy [*η* = 0.069, SE = 0.017, *p* < 0.001], depression [*η* = 0.107, SE = 0.012, *p* < 0.001], bipolar [*η* = 0.128, SE = 0.012, *p* < 0.001]), showing that rnn was able to predict subjects’ choices better than the other models.

The fact that lin and gql were better than ql and qlp is not unexpected; we showed in the previous section that the predictions from ql and qlp were inconsistent with the trial-by-trial behaviour of the subjects. On the other hand, the fact that rnn is better than lin and gql shows that there are some behavioural trends that even lin and gql failed to capture, although they were consistent with subjects’ choices according to the behavioural summary statistics. In the next sections, we use off-policy simulations of the models to uncover the additional behavioural trends that were captured by rnn.

### Off-policy simulations

**Fig 6:**
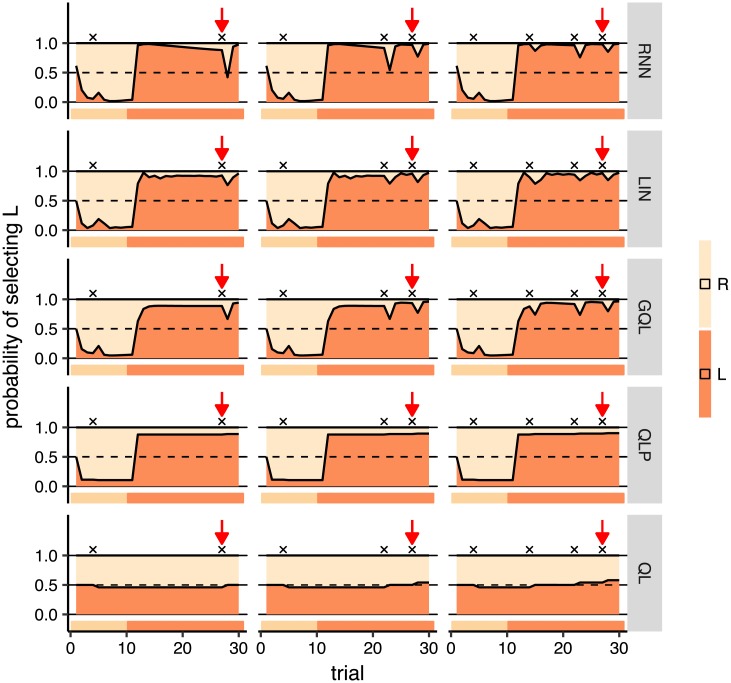
Off-policy simulations of all models for group healthy. Each panel shows a simulation of 30 trials (horizontal axis), and the vertical axis shows the predictions of each model on each trial. The ribbon below each panel shows the action which was fed to the model on each trial. In the first 10 trials, the action that the model received was R and in the next 20 trials it was L. Rewards are shown by black crosses (x) on the graphs. Red arrows point to the same trial number in all the simulations and are shown to compare changes in the predictions in that trial between different simulations. The sequence of rewards and actions fed to the model are the same for the panels in each column, but they are different across the columns. See text for the interpretation of the graph.

In an off-policy simulation, a model uses information about previous choices and rewards to make predictions about the next action. However, the actual next action used to simulate the model is not derived from these predictions and is derived in some other manner; notably, from human choices. In this way we can control the inputs the model receives and examine how they affect predictions. We were interested, in particular, in establishing how the predictions of the models were affected by the history of previous actions and rewards. As such, we designed a variety of inputs based on the behavioural statistics, fed them into the models, and recorded the predictions of each model in response to each input set (see S2 Text for more details on how simulation parameters were chosen). Simulations of the models are shown in each row of Fig 6 for the healthy group, in which each panel shows a separate simulation across 30 trials (horizontal axis). For trials 1-10, the action that was fed to the model was R (right action), and for trials 11-30 it was L, i.e., left action (the action fed into the model at each trial is shown in the ribbons below each panel). The rewards associated with these trials varied among simulations (the columns) and are shown by black crosses (x) in the graphs.

#### The effect of reward on choice

Focusing on the rnn simulations in Fig 6, it can be observed that earning a reward (shown by black crosses) caused a ‘dip’ in the probability of staying with an action, which showed a tendency to switch to the other action. This is consistent with the observation made in Fig 4 that the probability of switching increases after reward. We saw a similar pattern in gql, in which the contribution of action values to choices can be negative, i.e., higher values can lead to a lower probability of staying with an action (see S1 Text for more explanation). Similarly, in the lin model the effect of reward on the probability of the next action can be negative, which allowed this model to produce the observed pattern. The pattern, however, is reversed in the ql and qlp models, i.e., the probability of choosing an action increased after a reward due to an increase in action value, which is again consistent with the observations in Fig 4. The effects are rather small in these two models (and may not be clear for the qlp model), which is likely because the effect of reward needs to be non-zero in order to enable the model to direct choices toward the best action in the long run. At the same time the effect is in the wrong direction compared to the actual data and therefore it needs to be kept small to minimize the discrepancy of predictions with the actual actions after earning rewards.

**Fig 7:**
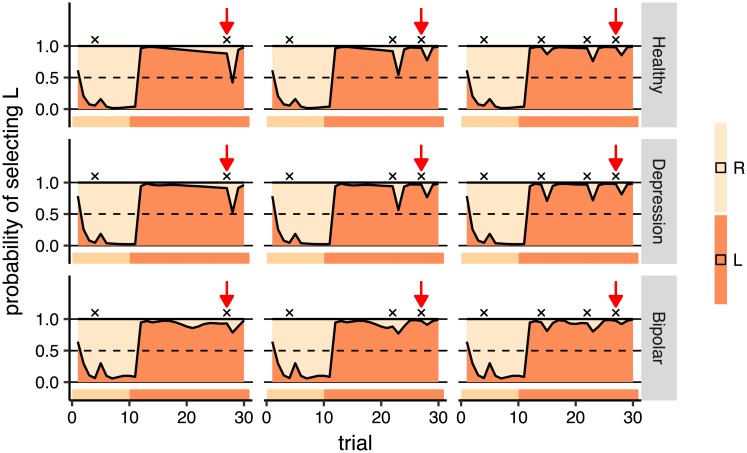
Off-policy simulations of rnn for all groups. Each panel shows a simulation of 30 trials (horizontal axis), and the vertical axis shows the predictions for each group on each trial. The ribbon below each panel shows the action which was fed to the model on each trial. In the first 10 trials, the action that the model received was R and in the next 20 trials it was L. Rewards are shown by black crosses (x) on the graphs, and the red arrows point to the same trial number in all the panels. See text for the interpretation of the graph. Note that the simulation conditions are the same as those shown in Fig 6, and the first row here (healthy group) is the same as the first row shown in Fig 6 which is shown again for comparison with the other groups.

The next observation was the effect of the previous reward on the probability of switching after a reward. First we focused on the rnn model and on the trials shown by red arrows in Fig 6. The red arrows point to the same trial number, but the number of rewards earned prior to the trial differed. As the figure shows, the probability of switching after reward was lower in the right-panel compared to the left and middle panels. The only difference between simulations is that, in the right panel, two more rewards were earned before the red arrow. Therefore, the figure shows that although the probability of switching was higher after reward, it got smaller as the number of rewards previously earned by an action increased. Indeed, this strategy made subjects switch more often from the inferior action, because rewards were sparse on that action, and switch less from the best action, because it was more frequently rewarded. This reconciles the observations made in Figs 3 and 4 that, although more responses were made on the better action, the probability of switching after reward was higher. Fig 7 shows the same simulations using rnn for all groups. Comparing the predictions at the red arrows for the depression and bipolar groups, we observed a pattern similar to the healthy group, although the differences were smaller in the bipolar group (see S11 Fig for the effect of the initialisation of the model).

**Fig 8:**
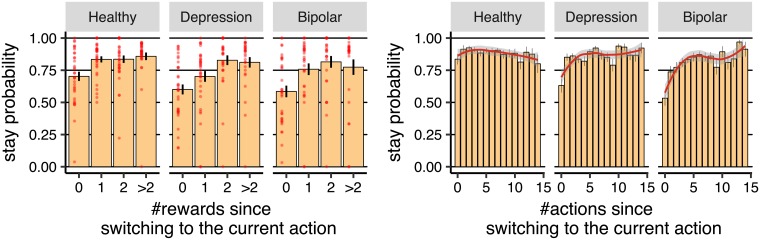
The effect of the history of previous rewards and actions on the future choices of the subjects. **(Left-panel)** The probability of staying with an action after earning reward as a function of the number of rewards earned since switching to the current action (averaged over subjects). Each red dot represents the data for one subject. **(Right-panel)** The probability of staying with an action as a function of the number of actions taken since switching to the current action. The red line was obtained using Loess regression (Local Regression), which is a non-parametric regression approach. The grey area around the red line represents the 95% confidence interval. Error-bars represent 1SEM.

The above observations are consistent with the pattern of choices in the empirical data shown in Fig 8-left panel, which depicts the probability of staying with an action after earning reward as a function of how many rewards were earned after switching to the action (a similar graph using on-policy simulation of rnn is shown in S13 Fig). In all three groups, the probability of staying with an action (after earning a reward) was significantly higher when more than two rewards were earned previously (>2) compared to when no reward was earned (healthy [*η* = 0.148, SE = 0.037, *p* < 0.001], depression [*η* = 0.188, SE = 0.045, *p* < 0.001], bipolar [*η* = 0.150, SE = 0.056, *p* = 0.012]), which is consistent with the behaviour of the rnn.

As shown in Fig 6, the gql model produced a pattern similar to rnn largely because this model tracks multiple values for each action, which allows this model to produce the ‘dip’s after reward, with a magnitude that is sensitive to the number of past rewards (see S1 Text for details). As such, it is not surprising that gql was consistent with the subjects’ choices with respect to the effects of immediate reward. Similarly, the lin model was able to produce this pattern, which is again expected as in this model the predicted probabilities are based on rewards earned several trials back (up to 18 trials back), which allows this model to learn about the effect of distant rewards on current actions. In this case, the rewards earned proximal to the action will have a negative effect on selecting the same action—generating the observed ‘dip’ in the probabilities—and distant rewards will have a positive effect on the probabilities of staying on the same action, making the size of the ‘dip’ sensitive to the number of rewards earned in the past (see S6, S7, S8, S9 Figs for the corresponding results for all of the groups based on the lin, gql, qlp and ql models respectively).

*The effect of repeating an action on choices*. Next, we looked at the effect of the history of actions on choices. Focusing on the rnn model in Fig 6, we can see that, in the first 10 trials, the predicted probability of taking R was higher than L; this then reversed over the next 20 trials. This implies that perseveration (i.e., sticking with the previously taken action) was an element of action selection. This is consistent with the fact that the qlp model (which has a parameter for perseveration) performed better than the ql model in the cross-validation statistics (see Fig 5); and, indeed, Fig 6 shows ql’s inability to reflect this characteristic. Note that it can be seen in Fig 3 that the probability of staying with an action was above 50% irrespective of whether a reward was earned on the previous trial or not. This does not, however, provide evidence for perseveration because the trials were not statistically independent. For example, in late training trials a subject might have discovered which action returns more reward on average and, therefore, stayed with that action irrespective of reward and so without necessarily relying on perseveration.

Focusing on rnn simulations in the left-panel of Fig 6, we observed that, after switching to action L (after trial 10), the probability of staying with that action gradually decreased; i.e., although there was a high chance the next action would be similar to the previous action, subjects developed a tendency to switch the longer they stayed with an action. To compare this pattern with the empirical data, we calculated the probability of staying with an action as a function of how many times the action had been taken since switching (Fig 8:right-panel; similar graphs for rnn, lin and gql on-policy simulations are shown in S13, S14 and S15 Figs respectively). As the figure shows, for the healthy group, the chance of staying with an action decreased as the action was repeated [*η* = −0.005, SE = 0.001, *p* = 0.006], which is consistent with the behaviour of rnn. With regard to the baseline models, going back to Fig 6, we did not see a similar pattern, although in gql there was a small decrement in the probability of staying with an action after earning the first reward.

*Symmetric oscillations between actions*. Next, we focussed on the rnn simulations in Fig 7 in depression and bipolar groups for which the gap between prediction accuracy of baseline models and rnn was largest. As can bee seen in the left-panels, after switching to action L (after trial 10), the probability of staying with that action gradually decreased in the depression group whereas, for the bipolar group, there was a ‘dip’ around 10 trials after the switching to action L (i.e., around trial 20), and then the policy became flat. With reference to the empirical data, as shown in Fig 8:right-panel, for the depression and bipolar groups, the probability of staying with an action immediately after switching to that action was around 50%—60% (shown by the bar at *x* = 0 in Fig 8:right-panel), i.e., there was a 40%–50% chance that the subject immediately switched back to the previous action. Based on this we expected to see a ‘dip’ in the simulations of the depression and bipolar groups in Fig 7 just after the switch to action L. This was not the case, pointing to an inconsistency between model predictions and the empirical data.

**Fig 9:**
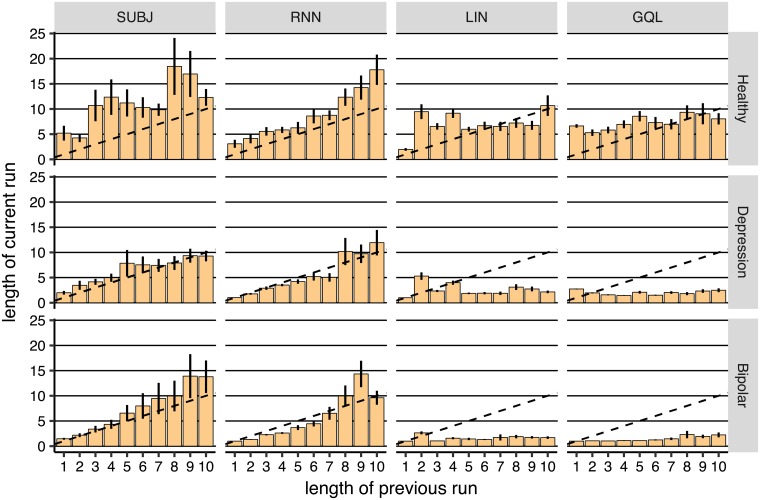
The median number of actions executed sequentially before switching to another action (run of actions) as a function of the length of the previous run of actions (averaged over subjects). The dotted line shows the points at which the length of the previous and the current run of actions were the same. Note that the median was used instead of the average to illustrate the most common ‘current run length’, instead of the average run length for each subject. The results for actual data are shown in subj column, and the remaining columns show the results using the on-policy simulations of the models in the task. Error-bars represent 1SEM.

However, Fig 7 is based on particular, artificial sequences of actions and rewards. To look more closely at the above effect, we defined a *run* of actions as a sequence of presses on a specifed button without switching to the other button. For example, if the executed actions were L, R, R, L, then the length of the first run was 1 (L), the length of the second run was 2 (R, R), and the length of the third run was 1 (L). Fig 9 shows the relationship between consecutive run length, i.e., the length of the current run of actions, as a function of the length of the previous run of actions in the empirical data. The graph show the empirical data (shown by subj) and on-policy model simulations. The dashed line in the figure indicates the points at which the current run length was the same as the previous run length. Being close to this line implies that subjects were performing symmetrical oscillations between the two actions, i.e., going back and forth between the two actions while performing an equal number of presses on each button. The data for subjects (shown in subj column) shows that, in the bipolar group, and to some extent in the depression group, a short run triggered a subsequent run of similar brevity (see S3, S4, and S5 Figs for raw empirical data). This implies that if a subject performed a run of length 1 that would initiate a sequence of oscillations between the two actions, keeping the stay probabilities low during short runs, consistent with what was seen at *x* = 0 in Fig 8:right-panel. This effect was not seen in the simulations shown in Fig 7, because the length of the previous run before switching to action L was 10 (there were 10 R actions), and therefore we should not expect the next run to be of length 1, nor should we have actually expected to see a ‘dip’ in policy just after the first switch.

**Fig 10:**
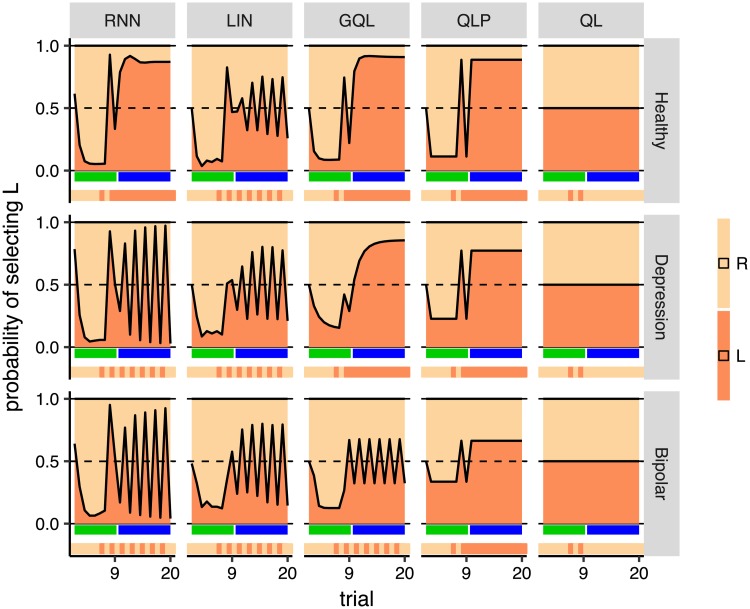
Mixed off-policy and on-policy simulations of the models. Each panel shows a simulation of 20 trials for which the first nine trials were off-policy and the subsequent trials were on-policy, during which the action with the highest probability was selected. Trials marked with green ribbons were off-policy (actions were fed to the model), whereas the trials marked with blue ribbons were on-policy (actions were selected by the model). The ribbon below each panel shows the actions that were fed to the model (for the first 9 trials), and the actions that were selected by the model (on the subsequent trials). During off-policy trials, the sequence of actions that was fed to the model was R, R, R, R, R, R, L, R, L. See the text for interpretation.

As shown in S12 Fig, the modal length of runs in the depression, and bipolar groups was 1 (around 17%, 37%, and 45% of runs were of length 1 in the healthy, depression, and bipolar groups respectively). Given this, and the specific pattern of oscillations in the depression and bipolar groups, our next question was whether, in the models, a run of length 1 triggered oscillations similar to those observed in the empirical data. We used a combination of off-policy and on-policy model simulations to answer this question; i.e., during the off-policy phase we forced the model to make an oscillation between the two actions, and then allowed the model to select between actions. We expected, in the healthy group, that the model would converge on one action, whereas, in the depression and bipolar groups, we expected the initial oscillations to trigger further switches. Simulations are presented in Fig 10, which shows that the sequence of actions fed to the model for the first 9 (off-policy) trials was:

R,R,R,R,R,R,L,R,L,

in which there were two oscillations at the tail of the sequence (R, L, R, L,). The rest of the actions (trials 10-20) were selected based on which action the model assigned the highest probability. Note that in on-policy simulations, actions were typically selected probabilistically according to the probabilities that a model assigned to each action. However, in the on-policy simulations presented in this section, in order to get consistent results across simulations actions were *not* selected probabilistically but were chosen based on which action achieved the highest prediction probability. As the simulation shows, at the beginning, the probability the model assigned to action R was high, but after feeding in the oscillations, the model predicted that the future actions would oscillate in the depression and bipolar groups, but not in the healthy group, consistent with what we expected to observe.

Therefore, rnn was able to produce symmetrical oscillations and its behaviour was consistent with the subjects’ actions. As Fig 10 shows, besides rnn, the lin and gql models were also able to produce length 1 oscillations to some extent (as shown for the bipolar group), which could explain why the prediction accuracy achieved by these models was significantly better than qlp in the bipolar and depression groups (Fig 5) in which length 1 oscillations were more common (see S2 Text for more details). However, as shown in Fig 9, lin and gql both failed to produce oscillations of longer lengths, whereas rnn was able to do so (Fig 9 rnn column). gql failed to produce the oscillations even if we increased the capacity of gql to track 10 different values for each action (see S16 Fig). Similarly, as the simulations show, the lin model was not able to produce these oscillations; such oscillations will likely require higher order interaction terms and, although these could be added to the LIN model in principal, in practise they will significantly complicate its transparent interpretation. This failing of the lin and gql models is particularly problematic in the depression and healthy groups, because these two groups tended to match the length of consecutive runs of actions. This could partly account for why the cross-validation statistics associated with rnn were significantly better than lin and gql for the depression and bipolar groups.

*Summary of off-policy simulations*. Firstly, we found that a rnn model was able to capture the immediate effect of rewards on actions (i.e., the ‘dip’ after rewards), as well as the effect of previous rewards on choices. gql and lin had a similar ability, which enabled them to reproduce the behavioural summary statistics shown in Figs 3 and 4. Baseline reinforcement-learning models (qlp and ql) failed to capture either trend. Secondly, rnn was able to capture how choices change as an action is chosen repeatedly and sequentially, and also the symmetrical oscillations between actions, neither of which could be detected by any of the baseline models.

### Diagnostic label prediction

In the previous sections we showed that there are several behavioural trends that baseline models failed to capture. Here we asked whether capturing such behavioural trends in this task is necessary to predict the diagnostic labels of the subjects. We used the leave-one-out cross-validation method based on which, in each run, one of the subjects in each group was withheld, and a rnn model was fitted to the rest of the group. This model, along with the versions of the same model fitted to all of the subjects in each of the other two groups, was used to predict the diagnostic label for the withheld subject. This prediction was based on which of the three models provided the best fit (lowest nlp) for that subject. As an example, assume that we are interested to predict the diagnostic label for a certain subject—say subject #10—in the depression group. We first trained a model using the data of all other subjects in the depression group, and we also trained two other models using the data of healthy and bipolar groups. Then, we evaluated which of these three models can better predict the actions of subject #10 in terms of nlp to predict the diagnostic label of the subject.

The results are reported in Table 1. Baseline random performance was near 33%. As the table shows, the highest performance was achieved for the healthy group of which 64% of subjects were classified correctly. A binomial test indicated that the proportion of correctly classified subjects in the healthy group was significantly different than the expected proportion of 0.336 based on random classification (*p* < 0.001 two-sided). On the other hand, in the depression group a significant portion of subjects were classified as healthy. A binomial test did not indicate that the proportion of correctly classified subjects in the depression and bipolar groups was significantly different than the expected proportion of 0.336 and 0.326, respectively, based on random classification (*p* > 0.1 two-sided).

**Table 1:** Prediction of diagnostic labels using rnn. The number of subjects in each true- and predicted-label. The numbers inside the parentheses are the percentage of subjects relative to the total number of subjects in each diagnostic group.

		predicted labels		
		healthy	depression	bipolar
true labels	healthy	22 (64%)	8 (23%)	4 (11%)
	depression	13 (38%)	16 (47%)	5 (14%)
	bipolar	9 (27%)	9 (27%)	15 (45%)

The overall correct classification rate of the model was 52%, whereas lin achieved 46% accuracy (S1 Table) and gql achieved 50% accuracy (S2 Table). We conclude that although lin and gql were unable to accurately characterize behavioural trends in the data, the group differences that were captured by lin and gql appeared sufficient to guide diagnostic label predictions.

## Discussion

We used a recurrent neural network to provide a framework for learning a computational model that can characterize human learning processes in decision-making tasks. Unlike previous work, the current approach makes minimal assumptions about these learning processes; we showed that this agnosticism is important in developing an appropriate explanation of the data. In particular, the rnn model was able to encode the melange of processes that subjects appeared to use to select actions; it was also able to capture differences between the psychiatric groups. These processes were largely inconsistent with conventional and tailored *Q*-learning models, and were also hidden in the overall performance of subjects on the task. This provided a clear example of how the currently proposed framework can outperform previous approaches.

In general, as we were able to show, this new approach improves upon previous methods from four standpoints: First, it provides a model that was able to predict subjects’ choices without requiring manual engineering; and to do so more accurately than baseline models on test data. Second, the framework contributes to computational modelling by providing a baseline for predictive accuracy; i.e., to the extent that other candidate models failed to generate the performance of rnn models, important and accessible behavioural trends would have been missed in the model structure. This is particularly important because the natural randomness of human choice in many scenarios makes it unclear whether the model at hand (e.g., a *Q*-learning model) has reached a limit as to how well those choices can be predicted, or whether it requires further improvements. Without other recourse, conventional treatments tend to relegate model mis-fit to irreducible randomness in choice. Third, based on the framework, a trained model can be regarded as representative of a group’s behaviour, which can then be interrogated in control conditions using off-policy simulations to gain insights into the learning processes behind subject’s choices. Finally, the framework can be used to predict the diagnostic labels of the subjects.

It might be possible to design different variants of *Q*-learning models (e.g., based on the analysis presented above) and obtain more competitive prediction accuracy. For example, although it is non-trivial, it is possible to design a new variant of gql able to track oscillatory behaviour such as that described here. Our aim was not to rule out this possibility, but rather to show that the framework can automatically extract learning features from subjects’ actions using learning to learn principles without requiring feature engineering in the models. This is even when those features were initially invisible in task performance metrics.

Our approach inherits these benefits from the field of neural networks [35], in which feature engineering has been significantly simplified across various domains [see also for example 36, for recent developments using probabilitic programming]. However, our approach also inherits the black-box nature of these neural networks, i.e., the lack of an interpretable working mechanism. This might not be an issue in some applications, such as the ones mentioned above; however, this needs to be addressed in other applications in which the aim of the study is actually to obtaining an interpretable working mechanism. Nevertheless, we were able to show that running controlled experiments on the model using off-policy simulations can provide significant insights into the processes that mediate subjects’ choices. An alternative method for interpreting the model is using gradients [37], which will be considered in future work. Interpreting neural networks is an active area of research in machine learning [e.g., 38], and the approach proposed here will benefit from further developments in this area.

In particular, although we found that off-policy simulations of the model could be used to gain insights into the model’s working mechanism, off-policy simulations need to be designed manually to determine inputs to the model. Here, we designed the initial off-policy simulations based on the specific questions and hypotheses that we were interested in testing and using overall behavioural statistics (Fig 6; S2 Text). However, an important aspect of the behavioural process, i.e., the tendency of subjects to oscillate between actions, was not visible in those simulations and, because of this, we had to design another set of inputs to investigate these oscillations (Fig 10). This shows that the choice of off-policy simulation can affect the interpretation of the model’s working mechanism. As such, although rnn can be trained automatically and without intuition into the behavioural processes behind actions [e.g., 39], designing off-policy simulations is not automated and requires manual hypothesis generation. Automating this process will require a method that generates representative inputs (and network outputs) that are clearly able to discriminate the differences between the psychiatric groups. The existence of adversarial examples in neural networks [40] suggests that this will not be as simple as using the networks to search explicitly for those input sequences that are the most discriminative—representativeness is also critical.

Recurrent neural networks have previously been used to study reward-related decision-making [13, 14], perceptual decision-making, performance in cognitive tasks, working-memory [15, 16, 17, 18, 19, 20], motor patterns, motor reach and timing [21, 22, 23, 24]. Typically, in these studies, an rnn is itself trained to perform the task. This is different from the current study in which the aim of training was to generate behaviour similar to the subjects’, even if that were to lead to poor performance on the task. One exception is the study of [21] in which a network was trained to generate outputs similar to electromyographic (EMG) signals recorded in behaving animals during a motor reach task. Interestingly, that study found that, even though the model was trained based purely on EMG signals, the internal activity of the model resembled the neural responses recorded from the subjects’ motor cortex. Indeed, we have recently used a similar approach to investigate whether brain activity during decision-making is related to network activity [37].

The fact that rnn performance was better than the baseline models can be attributed to two factors. Firstly, recurrent neural networks can potentially track a long history of previous events (such as rewards and actions) in order to predict the next actions. Evidence shows that humans tend to find patterns in the history of previous events (e.g., repetitions, alterations)—even if the events are generated randomly—and subsequently use those patterns to guide their choices and therefore it is important that a model be able to represent such inter-dependencies between current actions and past events [e.g., sequential effects; 41, 42, 43, 44, 45, 46, 47]. Secondly, such past influences might have a non-linear effect on current choices, and therefore it is important that the model be able to track higher-order statistics [48, 49] and non-linear effects. For example in the current study a linear logistic regression model (lin) was unable to reproduce the symmetrical oscillations between the actions whereas rnn could, which shows that non-linear dynamics are necessary to explain the current data.

With regard to predicting subjects’ diagnostic labels, it was perhaps not surprising to find that the model was unable to achieve a high level of classification accuracy. This is because there is a high level of heterogeneity in patients with the same diagnostic label. Heterogeneity, which is well understood in the wide variation in treatments and treatment outcomes in disorders like depression [e.g., 50], is likely also to be reflected in the differing learning and choice abilities of the subjects.

Given a set of models, different (approximate) Bayesian methods can be used for comparing different candidate models in order to find the model that has generated the data (or has the highest probability of being the model that has generated the data). This comparison can be achieved for example by calculating model-evidence, Bayes factors, exceedance probabilities [51], or using hierarchical Bayesian model comparison [52]. In some other settings, however, the aim is not just to compare a set of models, but to develop new models or to improve them in order to achieve a high out-of-sample prediction accuracy. A natural way to assess such prediction accuracy is to use cross-validation, that we used jointly with early stopping [30] to prevent the RNN from overfitting the data. Indeed, it has been suggested that, from a Bayesian perspective, the other quantities such as Akaike information criterion [AIC; 53], Deviance information criterion [DIC; 54, 55], and Watanabe-Akaike information criterion [WAIC; 56] can be viewed as approximations to different forms of cross-validation [57], which was directly calculated in the current study.

In the model fitting procedure used here, a single model was fitted to all of the subjects in each group, despite possible individual differences within a group. This was partly because we were interested in obtaining a single parameter set for making predictions for the subject withheld in the leave-one-out cross-validation experiments. Even if a mixed-effect model was fitted to the data, a summary of group statistics will be required to make predictions about a new subject. In other applications, one might be interested in estimating parameters for each individual (either network weights or the parameters of the reinforcement-learning models); in this respect using a hierarchical model fitting procedure would be a more appropriate approach, something that has been used previously for reinforcement-learning models [e.g., 4] and would be an interesting future step for rnn models.

Along the same lines, due to its rich set of parameters, a single rnn model might be able to learn about and detect individual differences (e.g., differences in the learning-rates of subjects) at an early stage of the task, and then use this information to make predictions about performance on later trials. For example, during the training phase (learning how humans learn), the model might learn that subjects have either a very high or a very low learning-rate. Then, when being evaluated in the actual learning task, the model can use observations from subjects’ choices on early trials to determine whether the learning-rate for that specific subject is high or low, and then utilise that information to make more accurate predictions in latter trials. Determining individual-specific traits in early trials of the task is presumably *not* part of the computational process occurring in the subject’s brain during the task, and is occurring in the model merely to make more accurate predictions. To the extent that the network learns such higher order structure, it is appealing, though difficult, to extract information about such heterogeneity from the recurrent state of the rnn. Of course, this implies that the (implicit) inferences that the rnn makes about the type of subject might be confounded with the (implicit) inferences that the rnn makes about the actual choices—thus it is a model that makes predictions about subjects’ choices using mechanisms that may not necessarily be competent computational models of the way that the subjects themselves make those choices.

## Materials and methods

### Ethics statement

The study was approved by the University of Sydney ethics committee (HREC #12812). Participants gave informed consent prior to participation in the study.

### Participants

34 uni-polar depression (depression), 33 bipolar (bipolar) and 34 control (healthy) participants (age, gender, IQ and education matched) were recruited from outpatient mental health clinics at the Brain and Mind Research Institute, Sydney, and the surrounding community. Participants were aged between 16 and 33 years. Exclusion criteria for both clinical and control groups were history of neurological disease (e.g. head trauma, epilepsy), medical illness known to impact cognitive and brain function (e.g. cancer), intellectual and/or developmental disability and insufficient English for neuropsychological assessment. Controls were screened for psychopathology by a research psychologist via clinical interview. Patients were tested under ‘treatment-as-usual’ conditions, and at the time of assessment, 77% of depressed and 85% of bipolar patients were taking medications (see Table 2 for breakdown of medication use). The study was approved by the University of Sydney ethics committee. Participants gave informed consent prior to participation in the study.

**Table 2:** Demographic and clinical characteristics of participants. **Means (SD)**. HDRS: Hamilton Depression Rating Scale; YMRS: Young Mania Rating Scale; SOFAS: Social and Occupational Functioning Scale; a: depression greater than healthy and bipolar, *p* < 0.05. b: bipolar greater than healthy, *p* < 0.05. c: healthy greater than depression and bipolar, *p* < 0.05.

	healthy (n = 34)	depression (n = 34)	bipolar (n = 33)
Demographics			
Gender (M:F)	15:19	15:19	9:24
Age in years	23.6 (4.3)	21.6 (2.5)	23.1 (4.4)
Predicted IQ	107.3 (7.5)	105.5 (7.9)	106.0 (7.4)
Eduation	14.3 (3.0)	13.3(1.9)	13.3 (2.4)
Symptoms and History			
Age of onset (years)	-	14.4 (3.8)	15.9 (4.7)
Duration of illness (years)	-	7.7 (4.3)	6.4 (3.3)
HDRS	1.5(2.0)	14.1 (7.2)^a^	8.9 (6.5)
YMRS	0.1 (0.4)	2.5 (5.4)	4.6 (5.8)^b^
SOFAS	91.0 (3.5)^c^	63.8 (9.2)	65.7 (13.7)
Medication			
Medicated	-	77%	85%
Anti-depressants	-	71%	41%
Mood stabilizers/Anti-convulsants	-	9%	73%
Lithium	-	0%	18%
Anti-psychotics	-	18%	33%
Anxiolytics	-	0%	3%
Motivation measures			
Hunger	6.5 (1.7)	6.0 (2.1)	6.0 (2.4)
Reward Pleasantness	3.1 (1.3)	2.0 (2.0)	2.6 (2.0)

Duration of illness indicates time since patient first experienced mental health problems, not time since diagnosis.

Demographics and clinical characteristics of the sample are presented in Table 2. Levene’s test indicated unequal variances for the HDRS [Hamilton Depression Rating Scale; 58], YMRS [Young Mania Rating Scale; 59], SOFAS [Social and Occupational Functional Scale; 60] and age, thus Welch’s statistic was used for these variables. A one-way ANOVA revealed no differences between groups in age [*F*(2, 98) = 2.48, *p* = 0.09], education [*F*(2, 98) = 1.76, *p* = 0.18], IQ [*F*(2, 94) = 0.47, *p* = 0.62] or gender (*χ*^2^ = 2.66, *p* = 0.27). There were differences in HDRS [*F*(2, 49.21) = 64.21, *p* < 0.001], YMRS [*F*(2, 43.71) = 12.57, *p* < 0.001], and SOFAS [*F*(2, 41.61) = 169.66, *p* < 0.001]. Bonferroni post-hoc comparisons revealed higher depression scores in depression group compared to bipolar and healthy groups, and higher depression in bipolar group compared to healthy group. Mania scores were significantly higher in the bipolar group compared to the healthy group. Both patient groups had significantly lower SOFAS scores compared to the healthy group, but did not differ from one another. Age of mental illness onset was younger in the depression group compared to the bipolar group [*t*(56) = −2.14, *p* = 0.04], however duration of illness did not differ significantly between groups [*t*(56) = 1.25, *p* = 0.22]. There were no differences between groups in pre-test hunger [*F*(2, 79) = 0.54, *p* = 0.59] or average snack rating [*F*(2, 79) = 2.53, *p* = 0.09].

### Task

The instrumental learning task (Fig 2) involved participants choosing between pressing a left or right button in order to earn food rewards (an M&M chocolate or a BBQ flavoured cracker). We refer to these two key presses as L and R for left and right button presses respectively. Fourteen healthy participants (41.2% of the group) and 13 bipolar participants (36.7% of the group) completed the task in an fMRI setting, using a 2 button Lumina response box. The remaining healthy and bipolar participants, and all depression participants, completed the task on a computer with a keyboard, where the “Z” and “?” keys were designated L and R. Although the performance of subjects was higher overall in the fMRI setting [*η* = 0.050, SE = 0.024, *p* = 0.041], the place in which the task was completed had no significant effect on how choices adjusted on a trial-by-trial basis, either on the probability of staying with the same action after earning a reward [*η* = 0.041, SE = 0.054, *p* = 0.45], or after no reward [*η* = 0.030, SE = 0.062, *p* = 0.627]), and, therefore, the data were combined.

During each block, one action was always associated with a higher probability of reward than the other. The best action was varied across blocks, and the probabilities varied between 0.25, 0.125, and 0.08. The probability of reward on the other action always remained at 0.05. Therefore, there were six pairs of reward probabilities and each was repeated twice. Participants were instructed to earn as many points as possible, as they would be given the concomitant number of M&Ms or BBQ flavoured crackers at the end of the session. After a non-rewarded response, a grey circle appeared in the centre of the screen for 250ms, whereas after a rewarded response the key turned green and an image of the food reward earned appeared in the centre of the screen for 500ms. A tally of accumulated winnings remained on the bottom of the screen for the duration of the task. The task began with a 0.25 contingency practice block and a pleasantness rating for each food outcome (-5 to +5). Responding was self-paced during the 12 blocks of training, each 40-s in length. On average participants completed 109.45, 114.91, 102.79 trials per block in healthy, depression, and bipolar groups respectively (see S9 Table for the average number of trials completed in each reward probability condition in each group). During inter-block intervals (12 seconds) the participants rated how causal each button was in earning rewards. These self-reports (causal ratings) are not used in the modelling analysis presented here.

### Computational models

#### Notation

The set of available actions is denoted by A and the total number of available actions is denoted by *N*_*a*_. Here A={L,R}, with L and R referring to left and right key presses respectively (*N*_*a*_ = 2). A set of subjects is denoted by S, and the total number of trials completed by subject s∈S over the whole task (all blocks) is denoted by $T_{s}$. $a_{t}^{s}$ denotes the action taken by subject *s* at trial *t*. The reward earned at trial *t* is denoted by *r*_*t*_, and we use *a*_*t*_ to refer to an action taken at time *t*, either by the subjects or the models (in simulations).

#### Recurrent neural network model (rnn)

**Architecture**. The architecture used is based on a recurrent neural network model (rnn) and is depicted in Fig 1. The model is composed of an lstm layer [Long short-term memory; 29] and an output softmax layer with two nodes (since there are two actions in the task). The inputs to the lstm layer are the previous action (*a*_*t*−1_ coded using one-hot transformation) and the reward received after taking action (*r*_*t*−1_ ∈ {0, 1}). The outputs of the softmax are probabilities of selecting each action, which are denoted by *π*_*t*_ (*a*; rnn) for action a∈A at trial *t*.

The lstm layer is composed of a set of lstm cells (*N*_*c*_ cells). Each cell is associated with (i) a cell state denoted by $c_{t}^{k}$ for cell *k* at time *t*, and (ii) cell output denoted by $h_{t}^{k}$ for cell *k* at time *t*. Cell states and outputs are initially zero and are updated after receiving each input. Let’s define ${\text{c}}_{t}={\left[c_{t}^{1},\ldots ,c_{t}^{N_{c}}\right]}^{T}$ as a vector containing cell states for all the cells at time *t* (${\text{c}}_{t}\in R^{N_{c}}$), and ${\text{h}}_{t}={\left[c_{t}^{1},\ldots ,c_{t}^{N_{c}}\right]}^{T}$ as a vector containing all the cell outputs at time (${\text{h}}_{t}\in R^{N_{c}}$). Furthermore, assume that **x**_*t*_ is a vector containing inputs to the network at time *t*, i.e., one-hot representation of *a*_*t*_ and *r*_*t*_ (${\text{x}}_{t}\in R^{N_{a}+1}$). The update rules for **c**_*t*_ and **h**_*t*_ are as follows:

$$\begin{array}{cc} {\text{f}}_{t}=\sigma (W_{f}{\text{x}}_{t}+U_{f}{\text{h}}_{t-1}+{\text{b}}_{f}) & \left({\text{f}}_{t}\in R^{N_{c}}\right) \end{array}$$ (1)

$$\begin{array}{cc} {\text{i}}_{t}=\sigma (W_{i}{\text{x}}_{t}+U_{i}{\text{h}}_{t-1}+{\text{b}}_{i}) & \left({\text{i}}_{t}\in R^{N_{c}}\right) \end{array}$$ (2)

$$\begin{array}{cc} {\text{o}}_{t}=\sigma (W_{o}{\text{x}}_{t}+U_{o}{\text{h}}_{t-1}+{\text{b}}_{o}) & \left({\text{o}}_{t}\in R^{N_{c}}\right) \end{array}$$ (3)

$$\begin{array}{cc} {\text{c}}_{t}={\text{f}}_{t}⊙{\text{c}}_{t-1}+{\text{i}}_{t}⊙\text{tanh}(W_{c}{\text{x}}_{t}+U_{c}{\text{h}}_{t-1}+{\text{b}}_{c}) & \left({\text{c}}_{t}\in R^{N_{c}}\right) \end{array}$$ (4)

$$\begin{array}{cc} {\text{h}}_{t}={\text{o}}_{t}⊙\text{tanh}\left({\text{c}}_{t}\right) & ({\text{h}}_{t}\in R^{N_{c}}), \end{array}$$ (5)

in which *σ* refers to the sigmoid function and ⊙ represents the element-wise Hadamard product. The parameters of the lstm layer include $W\in R^{N_{c}\times \left(N_{a}+1\right)}$, $U\in R^{N_{c}\times N_{c}}$, and $\text{b}\in R^{N_{c}}$.

The softmax layer takes outputs from the lstm layer as its inputs (**h**_*t*_) and provides the probability of selecting each action *π*_*t*_ (*a*; rnn). The parameter of the softmax layer is $V\in R^{N_{c}\times N_{a}}$, and therefore the parameters of the rnn model will be Θ = {*V*, *W*_*f*_, *W*_*i*_, *W*_*o*_, *W*_*c*_, *U*_*f*_, *U*_*i*_, *U*_*o*_, *U*_*c*_, **b**_*f*_, **b**_*i*_, **b**_*o*_, **b**_*c*_}.

**Training**. In the training phase (learning how humans learn), the aim is to train weights in the network so that the model learns to predict subjects’ actions given their past observations (i.e., it learns how *they* learn). This can be thought as a variant of a *learning-to-learn* process; albeit, more commonly, the learner is a human facing a series of learning tasks rather than a computer model trying to copy the human on a single task. For this purpose, the objective function for optimising weights in the network (denoted by Θ) for subject set S is,

$$L\left(\Theta ;\text{rnn}\right)=\sum_{s\in S}\sum_{t=1\ldots T_{s}}\text{log}\pi_{t}\left(a_{t}^{s};\text{rnn}\right),$$ (6)

where $a_{t}^{s}$ is the action selected by subjects *s* at trial *t*, and *π*_*t*_ (.; rnn) is the probability that model assigns to each action. Note that the policy is conditioned on the previous actions and rewards in each block of training; notation for this is omitted, for simplicity.

Models were trained using the maximum-likelihood (ML) estimation method,

$$\Theta_{\text{rnn}}^{\text{ML}}=\text{arg}\;\underset{\Theta }{\text{max}}L\left(\Theta ;\text{rnn}\right),$$ (7)

where Θ is a vector containing free-parameters of the model (in both lstm and softmax layers). The models were implemented in TensorFlow [61] and optimized using Adam optimizer [62]. Note that Θ was estimated for each group of subjects separately. Networks with different numbers *N*_*c*_ of lstm cells (*N*_*c*_ ∈ {5, 10, 20}) were considered, and the best model was selected using leave-one-out cross-validation (see below). Early stopping was used for regularization and the optimal number of training iterations was selected using leave-one-out cross-validation.

The total number of free parameters (in both the lstm layer and softmax layer) were 190, 580, and 1960 for the networks with 5, 10, and 20 lstm cells, respectively. In order to control for the effect of initialization of network weights on the final results, a single random network of each size (5, 10, 20) was generated, and was used to initialize the weights in the network.

After the training phase, the weights in the network were frozen and the trained model was used for three purposes: (i) cross-validation (see below), (ii) on-policy simulations and (iii) off-policy simulations. For cross-validation, the previous actions of the test subject(s) and the rewards experienced by the subject(s) were fed into the model, but unlike the training phase, the weights were not changing and we only recorded the prediction of the model about the next action. Note that even though the weights in the network were fixed, the output of the network changed from trial to trial due to the recurrent nature of these networks.

Due to the small sample size, we used the same set of subjects for testing the model and for the validation of model hyper-parameters (*N*_*c*_ and number of optimization iterations). That is, we calculated the prediction accuracy of the model in each group using cross-validation for different numbers of training iterations and different numbers of cells (S1 Fig) and chose the hyper-parameters (*N*_*c*_ and number of optimization iterations) that led to the highest performance (for the comparison with other models). Another alternative to this in-sample hyper-parameter selection was to use the data from two of the groups to obtain the optimal hyper-parameters (number of iterations/cells) for the other group. We found that the prediction accuracies obtained using these two alternatives were similar across the groups. The results reported in the paper are those derived using the estimations based on in-sample hyper-parameter estimations. S17 Fig shows cross-validation results using the alternative hyper-parameter estimation (using other groups for the estimations) and S10 Table shows the comparison of the cross-validation results obtained using the two methods.

Other than being used for calculating cross-validation statistics, trained models were used for on-policy and off-policy simulations (with frozen weights). In the on-policy simulations, the model received its own actions and earned rewards as inputs (instead of receiving the action selected by the subjects). In the off-policy simulations, the set of actions and rewards that the model received was fixed and predetermined. The details of these simulations are reported in the Results section.

**Model settings**. For the rnn model, leave-one-out cross-validation was used to determine the number of cells and optimisation iterations required for the rnn model to achieve the highest prediction accuracy. We found that the lowest mean negative log-probability (nlp) was achieved by 10 cells in the lstm layer and after 1100, 1200 optimisation iterations for the healthy and depression groups respectively whereas for the bipolar group the best nlp was achieved by 20 cells and 400 optimisation iterations (see S1 Fig). These settings were used for making predictions and simulations.

#### Baseline methods

For our baselines, we used ql, qlp and gql– which are variants and generalizations of *Q*-learning [31]—and lin, which is a logistic regression model.

ql *model*. After taking action *a*_*t*−1_ at time *t* − 1, the value of the action, denoted by *Q*_*t*_(*a*_*t*−1_), is updated as follows,

$$Q_{t}\left(a_{t-1}\right)=\left(1-\phi \right)Q_{t-1}\left(a_{t-1}\right)+\phi r_{t-1},$$ (8)

where *ϕ* is the learning-rate and *r*_*t*−1_ is the reward received after taking the action. Given the action values, the probability of taking action *a* ∈ {L, R} in trial *t* is:

$$\pi_{t}\left(a;\text{ql}\right)=\frac{e^{\beta Q_{t}\left(a\right)}}{\sum_{a^{\prime }\in A}e^{\beta Q_{t}\left(a^{\prime }\right)}},$$

where *β* > 0 is a free-parameter and controls the contribution of values to the choices (balance between exploration and exploitation). The free-parameters of this variant are *ϕ* and *β*. Note that the probability that the models predict for each action at trial *t* is necessarily based on the data *before* observing the action and reward at trial *t*. Further, since there are only two actions, we can write *π*_*t*_(L; ql) = 1 − *π*_*t*_(R; ql) = *σ*(*β*(*Q*_*t*_(L) − *Q*_*t*_(R))) where *σ*(⋅) is the standard logistic sigmoid.

Note that since here we are focused on modeling a bandit task, *Q*-values are represented as a function of actions and not states.

qlp *model*. This model is inspired by the fact that humans and other animals have a tendency to stick with the same action for multiple trials (i.e., perseverate), or sometimes to alternate between the actions [independent of the reward effects; 33]. We therefore call this model qlp, for *Q*-learning with perseveration. In it, action values are updated according to Eq 8 and so similarly to the ql model, but the probability of selecting actions is,

$$\pi_{t}\left(a;\text{qlp}\right)=\frac{e^{\beta Q_{t}\left(a\right)+k_{t}\left(a\right)}}{\sum_{a^{\prime }\in A}e^{\beta Q_{t}\left(a^{\prime }\right)+k_{t}\left(a^{\prime }\right)}},$$

where,

$$k_{t}\left(a\right)=\{\begin{array}{cc} \kappa & \text{if}\;a=a_{t-1}\\ 0 & \text{otherwise} \end{array}.$$ (9)

Therefore, there is a tendency to select the same action again on the next trial (if *κ* > 0) or switch to the other action (if *κ* < 0). In the specific case that *κ* = 0, the qlp model reduces to ql. Free-parameters are *ϕ*, *β*, *κ*.

gql *model*. As we will show in the results section, neither ql nor qlp fit the behaviour of the subjects in the task. As such, we aimed to develop a baseline model which could at least capture high-level behavioural trends, and we built a generalised *Q*-learning model, gql, to compare with rnn. In this variant, instead of learning a single action value for each action, the model learns *d* different values for each action, where the difference between the values learned for each action is that they are updated using different learning-rates. The action values for action *a* are denoted by **Q**(*a*), which is a vector of size *d*, and the corresponding learning-rates are denoted by vector Φ of size *d* (**0** ⪯ Φ ⪯ **1**). Based on this, the value of action *a*_*t*−1_ at trial *t* − 1 is updated as follows,

$${\text{Q}}_{t}\left(a_{t-1}\right)=\left(1-\Phi \right)⊙{\text{Q}}_{t-1}\left(a_{t-1}\right)+r_{t-1}\Phi ,$$ (10)

where as mentioned before ⊙ represents the element-wise Hadamard product. For example, if *d* = 2, and Φ = [0.1, 0.05], then the model will learn two different values for each action (L, R actions) with one of the values updated using a learning-rate of 0.1 and the other updated using a learning-rate of 0.05. In the specific case that *d* = 1, the above equation reduces to Eq 8 used in ql and qlp models, in which only a single value is learned for each action.

In the qlp model, the current action is affected by the last taken action (perseveration). This property is generalised in the gql model by learning the history of previously taken actions instead of just the last action. These action histories are denoted by **H**(*a*) for action *a*. **H**(*a*) is a vector of size *d*, and each entry of this vector tracks the tendency of taking action *a* in the past, i.e., if an element of **H**(*a*) is close to one it means that action *a* was taken frequently in the past and being close to zero implies that the action was taken rarely. In similar fashion to action values, for each action *d* different histories are tracked, each of which is modulated by a separate learning-rate. Learning-rates are represented in vector Ψ of size *d* (**0** ⪯ Ψ ⪯ **1**). Assuming that action *a*_*t*−1_ was taken at trial *t* − 1, **H**(*a*) updates as follows,

$${\text{H}}_{t}\left(a\right)=\{\begin{array}{cc} \left(1-\Psi \right)⊙{\text{H}}_{t-1}\left(a\right)+\Psi & \text{if}\;a=a_{t-1}\\ \left(1-\Psi \right)⊙{\text{H}}_{t-1}\left(a\right) & \text{otherwise} \end{array}.$$ (11)

Intuitively, according to the above equation, if action *a* was taken on a trial, **H**(*a*) increases (the amount of increase depends on the learning-rate of each entry), and for the rest of the actions, **H**(other actions) will decrease (again the amount of decrement is modulated by the learning rates). For example, if *d* = 2, and Ψ = [0.1, 0.05], it means that for each action two choice tendencies will be learned, one of which is updated by rate 0.1 and the other one by rate 0.05.

Having learned **Q**(*a*) and **H**(*a*) for each action, the next question is how are they combined to guide choice. *Q*-learning models assume that the contribution of values to choices is modulated by parameter *β*. Here, since the model learns multiple values for each action, we assume that each value is weighted by a separate parameter, denoted by vector **B** of size *d*. Similarly, in the qlp model the contribution of perseveration to choices is controlled by parameter *κ*, and here we assume that parameter **K** modulates the contribution of previous actions to the current choice. Based on this, the probability of taking action *a* at trial *t* is,

$$\pi_{t}^{\prime }\left(a;\text{gql}\right)=\frac{e^{\text{B}\cdot {\text{Q}}_{t}\left(a\right)+\text{K}\cdot {\text{H}}_{t}\left(a\right)}}{\sum_{a^{\prime }\in A}e^{\text{B}\cdot {\text{Q}}_{t}\left(a^{\prime }\right)+\text{K}\cdot {\text{H}}_{t}\left(a^{\prime }\right)}},$$

where “⋅” operator refers to the inner product. Here, we also add extra flexibility to the model by allowing values to interact with the history of previous actions in influencing choices. For example, if *d* = 2, we allow the two learned values for each action to interact with the two learned action histories of each action, leading to four interaction terms, and the contribution of each interaction term to choices is determined by a matrix **C** of size *d* × *d* (*d* = 2 in this example),

$$\pi_{t}\left(a;\text{gql}\right)=\frac{e^{\text{B}\cdot {\text{Q}}_{t}\left(a\right)+\text{K}\cdot {\text{H}}_{t}\left(a\right)+{\text{H}}_{t}\left(a\right)\cdot \text{C}\cdot {\text{Q}}_{t}\left(a\right)}}{\sum_{a^{\prime }\in A}e^{\text{B}\cdot {\text{Q}}_{t}\left(a^{\prime }\right)+\text{K}\cdot {\text{H}}_{t}\left(a^{\prime }\right)+{\text{H}}_{t}\left(a^{\prime }\right)\cdot \text{C}\cdot {\text{Q}}_{t}\left(a^{\prime }\right)}},$$ (12)

The free-parameters of this model are Φ, Ψ, **B**, **K**, and **C**. In this paper we use models with *d* = 1, 2, 10, which have 5, 12 and 140 free parameters respectively. We used *d* = 2 for the results reported in the main text, since this model setting was able to capture several behavioural trends while still being interpretable. The results using *d* = 1, 10 are reported in the supplementary materials to illustrate the models’ capabilities in extreme cases.

lin *model*. The probability of taking each action is determined by a history past rewards and actions—up to *J* trials back—using a linear logistic regression model,

$$\text{log}\frac{\pi_{t}\left(a=\text{L};\text{lin}\right)}{\pi_{t}\left(a=\text{R};\text{lin}\right)}=\{\begin{array}{cc} \mu_{0}+\sum_{j=1}^{J}\mu_{j}a_{t-j}+\gamma_{j}r_{t-j}+\zeta_{j}a_{t-j}r_{t-j} & J>0\\ \mu_{0} & J=0 \end{array}.$$ (13)

Parameter *J* was selected using cross-validation (S2 Fig), which indicated that *J* = 18 provides the best nlp mean, and therefore the model with *J* = 18 was used in the analyses presented in the paper.

*Objective function*. The objective function for optimising the models was the same as the one chosen for rnn,

$$L\left(\Theta ;M\right)=\sum_{s\in S}\sum_{t=1\ldots T_{s}}\text{log}\pi_{t}\left(a_{t}^{s};M\right),M\in \left\{\text{ql},\text{qlp},\text{gql},\text{lin}\right\},$$ (14)

where, as mentioned before, $a_{t}^{s}$ is the action selected by subject *s* at trial *t*, and $\pi_{t}\left(.;M\right)$ is the probability that model M assigns to each action. Models were trained using the maximum-likelihood estimation method,

$$\Theta_{M}^{\text{ML}}=\text{arg}\;\underset{\Theta }{\text{max}}L\left(\Theta ;M\right),$$ (15)

where Θ is a vector containing the free-parameters of the models. Optimizations for all models except lin, were performed using Adam optimizer [62], and using the automatic differentiation method provided in TensorFlow [61]. The free-parameters with limited support (*ϕ*, *β*, Φ, Ψ) were transformed to satisfy the constraints. For lin model, we used ‘glm’ method in R [63] with ‘binomial’ link function to estimate the parameters and to make predictions.

#### Performance measures

Two different measures were used for quantifying the predictive accuracy of the models. The first measure is the average log-probability of the models’ prediction for the actions taken by subjects. For a group of subjects denoted by S, we define negative log-probability (nlp) as follows:

$$\text{nlp}=-\frac{\sum_{s\in S}\sum_{t=1\ldots T_{s}}\text{log}\pi_{t}\left(a_{t}^{s};M\right)}{\sum_{s\in S}T_{s}},M\in \left\{\text{rnn},\text{lin},\text{gql},\text{ql},\text{qlp}\right\}.$$ (16)

The other measure is the percentage of actions predicted correctly,

$$\%\text{correct}=\frac{\sum_{s\in S}\sum_{t=1\ldots T_{s}}⟦\text{arg}\;{\text{max}}_{a}\pi_{t}\left(a;M\right)=a_{t}^{s}⟧}{\sum_{s\in S}T_{s}},$$ (17)

where ⟦.⟧ denotes the indicator function. Unlike, ‘%correct’, nlp takes the probabilities of predictions into account instead of making binary predictions for the next action. In this way, if the models are certain about wrong predictions nlp performance gets penalized, and it gets credit if the models are certain about a correct prediction.

#### Model selection

Leave-one-out cross-validation was used for comparing different models. At each round, one of the subjects was withheld and the model was trained using the remaining subjects; the trained model was then used to make predictions about the withheld subject. The withheld subject was rotated in each group, yielding 34, 34 and 33 prediction accuracy measures in the healthy, depression, and bipolar groups respectively.

### Statistical analysis

For the analysis we performed hierarchical linear mixed-effects regression using the lme4 package in R [64] and obtained *p*-values for regression coefficients using the lmerTest package [65]. Hierarchical mixed-effects models involve random-effects and fixed-effects. Fixed-effects are of primary interest and are estimated directly (fixed-effects estimates are denoted by *η*). Random-effects specify different levels at which the data is collected (e.g., different subjects), i.e., fixed-effects are nested within random-effects in a hierarchical manner. Specific fixed-effects and random-effects used for each analysis are mentioned below for each analysis. For each test we report parameter estimate (*η*), standard error (SE), and *p*-value.

For the analysis presented in section ‘Performance in the task’ the intercept term was the random-effect at the subject level; action (low reward probability = 0, high reward probabilities = 1) was the fixed-effect; the dependent variable was the probability of selecting the action. For the second set of analyses in this section, the intercept term was the random-effect at the subject level; and action (low reward probability = 0, high reward probabilities = 1), groups (healthy = 0, depression = 1/bipolar = 1) and their interaction were fixed-effects; the dependent variable was the probability of selecting the action.

In the analysis in section ‘The immediate effect of reward on choice’ the intercept was the random-effect at the subject level; whether reward was earned on the previous trial was the fixed-effect and the probability of staying on the same action was the dependent variable.

In the analysis presented in section ‘Action prediction’ the intercept term was the random-effect at the cross-validation fold level; model (gql = 1, qlp = 0 for the first analysis and lin = 1, rnn = 0 for the second analysis) was the fixed-effect. nlp was the dependent variable.

In the analysis presented in section ‘The effect of reward on choice’ the intercept was the random-effect at the subject level; whether zero rewards or more than two rewards were earned previously was fixed-effect. The dependent variable was the probability of staying with an action.

In the analysis presented in section ‘Task’, the intercept term was the random-effect at the group level (healthy or bipolar), and the mode of task completion (fMRI setting = 1, computer = 0) was the fixed-effect; the probability of selecting the better key was the dependent variable.

In the analysis presented in section ‘The effect of reward on choice’ the intercept was the random-effect at the subject level; the number of times that an action was repeated since switching to the action was the fixed-effect (between zero to 15 times). The dependent variable was the probability of staying with an action. Note that in Fig 8:right-panel in this section, to be consistent with off-policy simulations, only trials on which (i) subjects did not earn a reward on that trial, and (ii) subjects did not earn a reward since switching to the current action, were included in the graph.

For Loess regression [66], ‘loess’ method in R was used [63].

## Supporting information

S1 Text Behavioural analysis using gql. (PDF)

S2 Text The choice of off-policy settings. (PDF)

S3 Text Analysis of randomness of choices. (PDF)

S1 Fig Cross-validation results for different numbers of cells and optimization iterations. **(Top-panel)** Percentage of actions predicted correctly averaged over leave-one-out cross-validation folds. **(Bottom-panel)** Mean nlp averaged over cross-validation folds. Error-bars represent 1SEM.
(PDF)

S2 Fig Cross-validation results for the lin model as a function of number of trials back (*J*). **(Left-panel)** nlp (negative log-probability) averaged across leave-one-out cross-validation folds. Lower values are better. **(Right-panel)** Percentage of actions predicted correctly averaged over cross-validation folds. Error-bars represent 1SEM.
(PDF)

S3 Fig Choices of the healthy group. Each row shows the choices of a subject across different blocks (12 blocks).
(PDF)

S4 Fig Choices of the depression group. Each row shows the choices of a subject across different blocks (12 blocks).
(PDF)

S5 Fig Choices of the bipolar group. Each row shows the choices of a subject across different blocks (12 blocks).
(PDF)

S6 Fig Off-policy simulations of lin. Each panel shows a simulation for 30 trials (horizontal axis), and the vertical axis shows the predictions for each group on each trial. The ribbon below each panel shows the action which was fed to the model on each trial. In the first 10 trials, the action that the model received was R and in the next 20 trials it was L. Rewards are shown by black crosses (x) on the graphs. See text for the interpretation of the graph. Note that the simulation conditions are same as those depicted in Figs 7 and 6.
(PDF)

S7 Fig Off-policy simulations of gql (*d* = 2). Each panel shows a simulation for 30 trials (horizontal axis), and the vertical axis shows the predictions for each group on each trial. The ribbon below each panel shows the action which was fed to the model on each trial. In the first 10 trials, the action that the model received was R and in the next 20 trials it was L. Rewards are shown by black crosses (x) on the graphs. See text for the interpretation of the graph. Note that the simulation conditions are the same as those depicted in Figs 7 and 6.
(PDF)

S8 Fig Off-policy simulations of qlp. Each panel shows a simulation for 30 trials (horizontal axis), and the vertical axis shows the predictions for each group on each trial. The ribbon below each panel shows the action which was fed to the model on each trial. In the first 10 trials, the action that the model received was R and in the next 20 trials it was L. Rewards are shown by black crosses (x) on the graphs. See text for the interpretation of the graph. Note that the simulation conditions are the same as those depicted in Figs 7 and 6.
(PDF)

S9 Fig Off-policy simulations of ql. Each panel shows a simulation for 30 trials (horizontal axis), and the vertical axis shows the predictions for each group on each trial. The ribbon below each panel shows the action which was fed to the model on each trial. In the first 10 trials, the action that the model received was R and in the next 20 trials it was L. Rewards are shown by black crosses (x) on the graphs. See text for the interpretation of the graph. Note that the simulation conditions are the same as those depicted in Figs 7 and 6.
(PDF)

S10 Fig Off-policy simulations of gql with *d* = 1. Each panel shows a simulation for 30 trials (horizontal axis), and the vertical axis shows the predictions for each group on each trial. The ribbon below each panel shows the action which was fed to the model on each trial. In the first 10 trials, the action that the model received was R and in the next 20 trials it was L. Rewards are shown by black crosses (x) on the graphs. See text for the interpretation of the graph. Note that the simulation conditions are the same as those depicted in Figs 7 and 6.
(PDF)

S11 Fig The effect of the initialisation of the network on the off-policy simulations of rnn. The simulation conditions are the same as those depicted in Figs 7 and 6. Here, 15 different initial networks were generated and optimised and the policies of the models on each trial were averaged. The grey ribbon around the policy shows the standard deviation of the policies. Each panel shows a simulation for 30 trials (horizontal axis), and the vertical axis shows the predictions of each model on each trial. The ribbon below each panel shows the action which was fed to the model on each trial. In the first 10 trials, the action that the model received was R and in the next 20 trials it was L. Rewards are shown by black crosses (x) on the graphs. See text for the interpretation of the graph.
(PDF)

S12 Fig Percentage of each run of actions relative to the total number of runs for each subject. Percentage of each length of run of actions relative to the total number of runs in each subject (averaged over subjects). Red dots represent data for each subject, and error-bars represent 1SEM.
(PDF)

S13 Fig rnn simulations. The graph is similar to Fig 8 but using data from rnn simulations (on-policy). **(Left-panel)** Probability of staying with an action after earning reward as a function of the number of actions taken since switching to the current action (averaged over subjects). Each red dot represents the data for each subject. **(Right-panel)** Probability of staying with an actions as a function of the number of actions taken since switching to the current action. The red line was obtained using Loess regression (Local Regression), which is a non-parametric regression approach. The grey area around the red line represents 95% confidence interval. Error-bars represent 1SEM.
(PDF)

S14 Fig lin simulations. The graph is similar to Fig 8 but using data from lin simulations (on-policy). **(Left-panel)** Probability of staying with an action after earning reward as a function of the number of actions taken since switching to the current action (averaged over subjects). Each red dot represents the data for each subject. **(Right-panel)** Probability of staying with an actions as a function of the number of actions taken since switching to the current action. The red line was obtained using Loess regression (Local Regression), which is a non-parametric regression approach. The grey area around the red line represents 95% confidence interval. Error-bars represent 1SEM.
(PDF)

S15 Fig gql simulations (*d* = 2). The graph is similar to Fig 8 but using data from gql simulations with *d* = 2 (on-policy). **(Left-panel)** Probability of staying with an action after earning reward as a function of the number of actions taken since switching to the current action (averaged over subjects). Each red dot represents the data for each subject. **(Right-panel)** Probability of staying with an actions as a function of the number of actions taken since switching to the current action. The red line was obtained using Loess regression (Local Regression), which is a non-parametric regression approach. The grey area around the red line represents 95% confidence interval. Error-bars represent 1SEM.
(PDF)

S16 Fig gql simulations (*d* = 10). The graph is similar to Fig 9 but using data from gql simulations with *d* = 10 (on-policy). Median number of actions executed in a row before switching to another action (run of actions) in each subject as a function of the length of the previous run of actions (averaged over subjects). The dotted line shows the points at which the length of the previous and current runs are the same. Note that the median rather than the average was because we aimed to illustrate the most common ‘length of current run’, instead of average run length in each subject. Error-bars represent 1SEM.
(PDF)

S17 Fig Cross-validation results. **(Left-panel)** nlp (negative log-probability) averaged across leave-one-out cross-validation folds. Lower values are better. **(Right-panel)** Percentage of actions predicted correctly averaged over cross-validation folds. Note that the difference between this figure and Fig 5 is that in Fig 5 hyper-parameters were obtained using in-sample estimations but here we used the data from two of the groups to obtain the optimal hyper-parameters (number of iterations/cells) for the other group. See text for more information.
(PDF)

S1 Table Prediction of diagnostic labels using lin. Number of subjects for each true- and predicted-label. The numbers inside parentheses are the percentage of subjects relative to the total number of subjects in each diagnostic group.
(PDF)

S2 Table Prediction of diagnostic labels using gql (*d* = 2). Number of subjects for each true- and predicted-label. The numbers inside parentheses are the percentage of subjects relative to the total number of subjects in each diagnostic group.
(PDF)

S3 Table Estimated parameters for ql model. (PDF)

S4 Table Estimated parameters for qlp model. (PDF)

S5 Table Estimated parameters for gql model with *d* = 2. (PDF)

S6 Table Negative log-likelihood for each model optimized over all the subjects in each group. (PDF)

S7 Table Negative log-likelihood for each model. For rnn a single model was fitted to the whole group using ML estimation. For baseline methods (gql, qlp, and ql), a separate model was fitted to each subject, and the reported number is the sum of negative log-likelihoods over the whole group.
(PDF)

S8 Table Mean and standard deviation of negative log-likelihood for rnn over 15 different initialisations of the model and optimised over all the subjects in each group. (PDF)

S9 Table Average number of trials in each pair of reward probabilities in each group. (PDF)

S10 Table Mean of nlp derived using in-sample hyper-parameter estimation (in-sample) and using the data of other groups (other-groups). (PDF)

S1 Data Supporting data. (ZIP)
