# Supplementary material for: Models that learn how humans learn: The case of decision-making and its disorders
Source: PLoS Comput Biol. 2019 Jun 11;15(6):e1006903. doi: 10.1371/journal.pcbi.1006903 (PMC6588260; doi:10.1371/journal.pcbi.1006903)
Supplement: S1 Text — (PDF) [file pcbi.1006903.s001.pdf]

S1 Behavioural analysis using GQL

In the GQL simulations presented in Figure 6, we observed that earning a reward (shown by black crosses) causes a 'dip' in the probability of staying with an action, but that the 'dip' decreases in magnitude with the number of recent rewards. Neither of these phenomena arises from QL and QLP; GQL captures them because it learns that the values of each action are updated at two different rates, positively with reward at a slow rate (0.145); and negatively with reward a fast rate (0.815; see Table S5).

The 'dip' implies a tendency to switch to the other action. This is consistent with a phenomenon that is apparent in Figure 4, namely that the probability of switching increases after rewards. The 'dip' is produced by the fast updating process with its negative reward contribution.

However, the fast process is also fast to forget, and so has an influence that cannot accumulate. The slow process ultimately exerts a stronger effect; and furthermore accumulates over multiple rewards in a way that the fast process cannot accomplish.

Based on this, allowing the model to track two different values for each action is important, and the model will not be able to produce this behaviour if it tracks only one value for each action ($d = 1$) as shown in Figure S10.
