# Supplementary material for: Models that learn how humans learn: The case of decision-making and its disorders
Source: PLoS Comput Biol. 2019 Jun 11;15(6):e1006903. doi: 10.1371/journal.pcbi.1006903 (PMC6588260; doi:10.1371/journal.pcbi.1006903)
Supplement: S2 Text — (PDF) [file pcbi.1006903.s002.pdf]

S2 The choice of off-policy settings

In the simulations shown in Figure 6, action R is fed into the model for the first 10 trials; then a switch is made to action L. This is based on the fact that in the empirical data, the average length of staying with an action (when one reward is earned in the middle of the 'run' of the action) is 9.8. The first, second and third rewards in Figure 6 are delivered after an action was taken 4, 12, and 17 times respectively. This is based on the fact that in the empirical data, the average number of key-presses in order to earn the first, second and third rewards is 4.07, 11.6, and 17.4 respectively.

In the simulations shown in Figure 10, the reason for adding leading R before oscillations is to show that the models do not oscillate all the time, but only after they are fed with oscillations. Indeed, QLP is in principle able to produce 1-step oscillations (singe-action runs) by assigning a negative weight to the perseveration parameter, i.e., instead of the model having a tendency to stay on the previously selected action, it will have a tendency to switch from this action. However, under this condition the model will keep oscillating between the actions from trial 1, implying that it can only produce runs of length 1 no matter what the length of the previous run of actions was, which is inconsistent with the empirical data presented in Figure 9.
