# Supplementary material for: Models that learn how humans learn: The case of decision-making and its disorders
Source: PLoS Comput Biol. 2019 Jun 11;15(6):e1006903. doi: 10.1371/journal.pcbi.1006903 (PMC6588260; doi:10.1371/journal.pcbi.1006903)
Supplement: S3 Text — (PDF) [file pcbi.1006903.s003.pdf]

S3 Analysis of randomness of choices

The performance of 0.5 in Figure 3 implies that on average the two actions have been selected equally. However, the behaviour of performance around 0.5 does not necessarily imply that actions are chosen randomly. For example, a subject might constantly switch between the two keys (L R L R . . .), which will lead to a 0.5 performance, but with actions that are far from random. The results of a 'Wald-Wolfowitz runs test' (using 'runs.test' in R 'randtests' package described in Caeiro and Mateus, 2014), indicated that the behaviour of 96 of the subjects failed this test, i.e., was considered not to be random (p-value$<$0.001). Of the remaining 5 subjects, only one was given an incorrect diagnostic label prediction by our method (the actual label was Bipolar when it was identified as Depression).
