# Supplementary figures and images for: Models that learn how humans learn: The case of decision-making and its disorders
Source: PLoS Comput Biol. 2019 Jun 11;15(6):e1006903. doi: 10.1371/journal.pcbi.1006903 (PMC6588260; doi:10.1371/journal.pcbi.1006903)

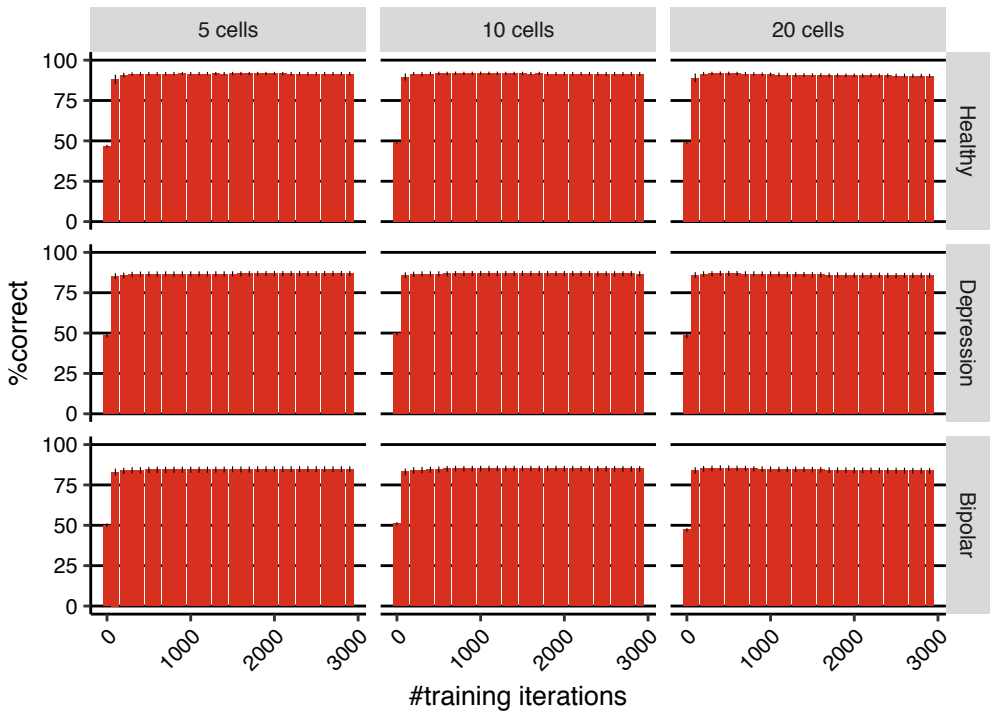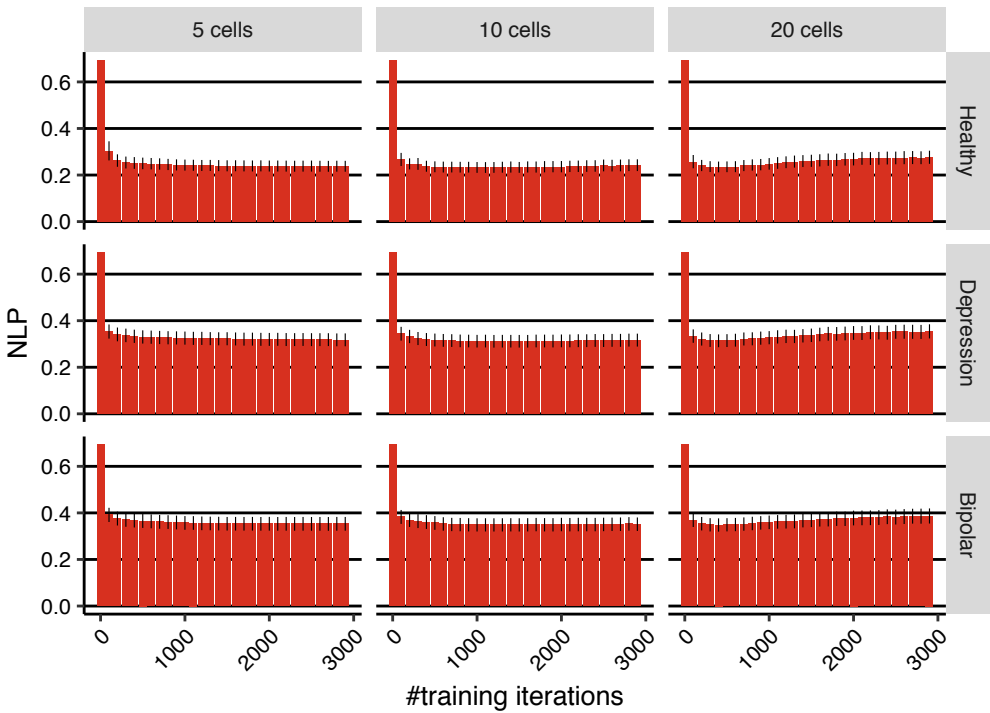

Supplement: S1 Fig — (Top-panel) Percentage of actions predicted correctly averaged over leave-one-out cross-validation folds. (Bottom-panel) Mean nlp averaged over cross-validation folds. Error-bars represent 1SEM. (PDF) [file pcbi.1006903.s004.pdf]

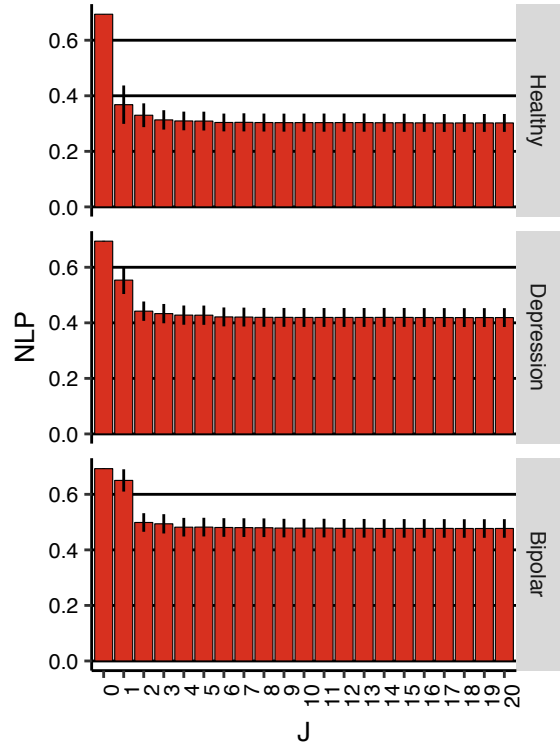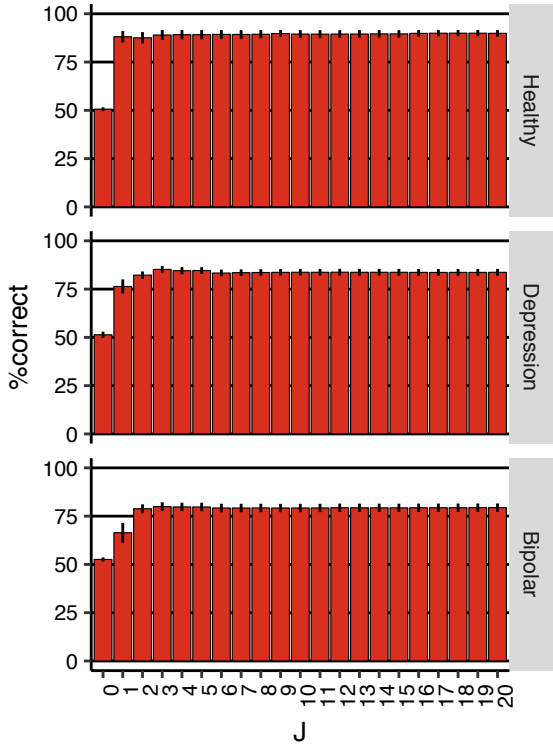

Supplement: S2 Fig — (Left-panel) nlp (negative log-probability) averaged across leave-one-out cross-validation folds. Lower values are better. (Right-panel) Percentage of actions predicted correctly averaged over cross-validation folds. Error-bars represent 1SEM. (PDF) [file pcbi.1006903.s005.pdf]

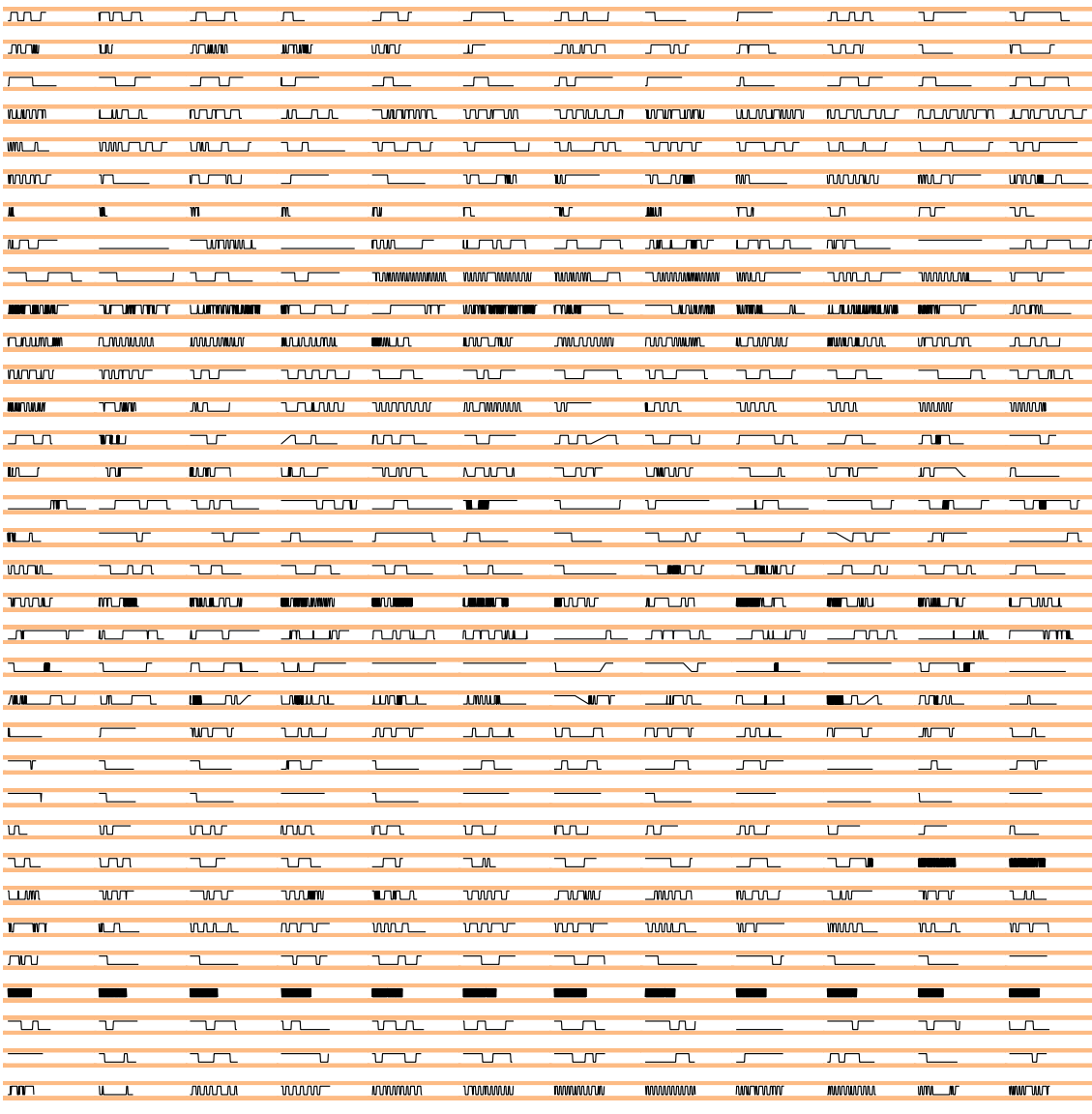

Supplement: S3 Fig — Each row shows the choices of a subject across different blocks (12 blocks). (PDF) [file pcbi.1006903.s006.pdf]

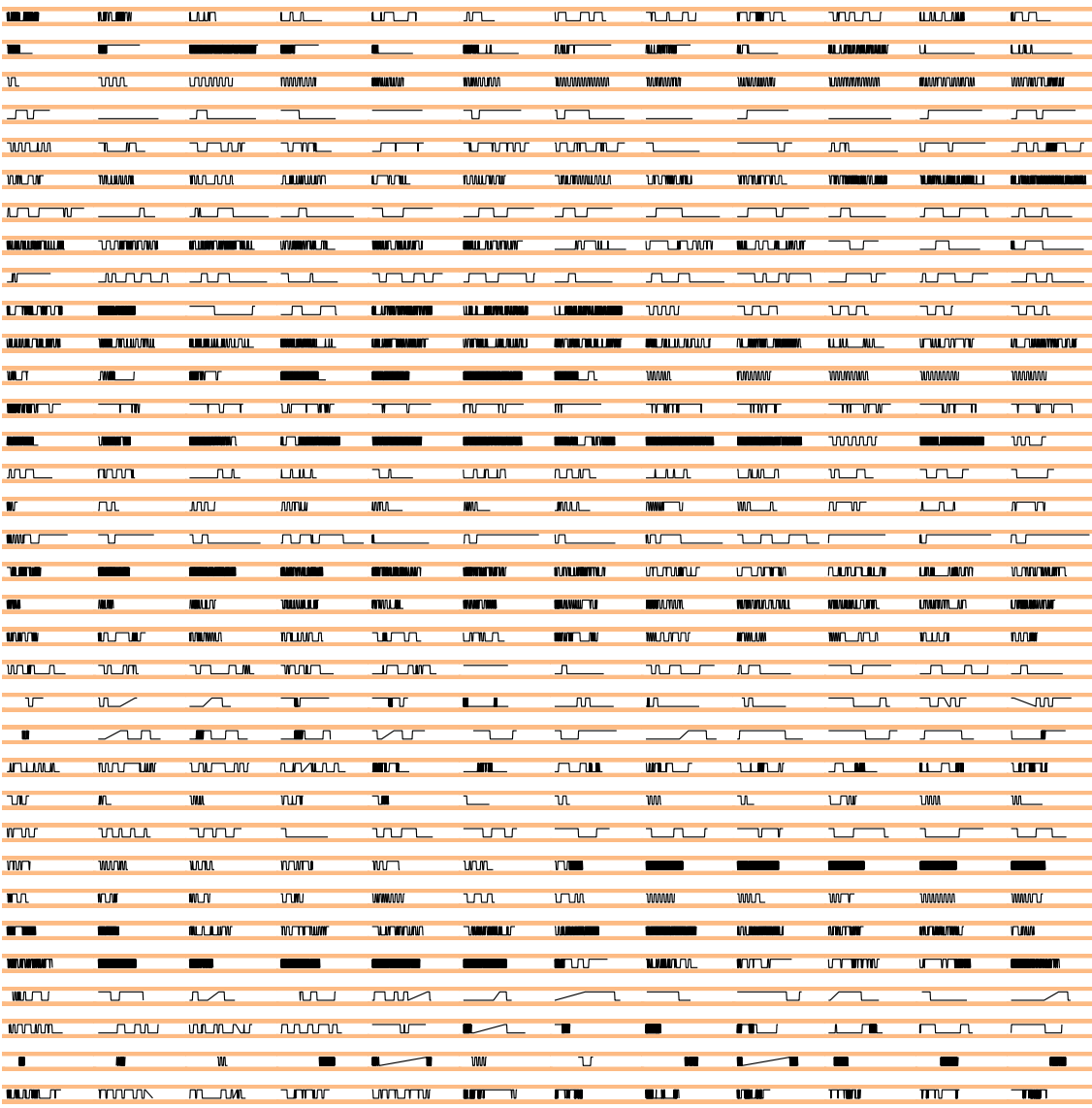

Supplement: S4 Fig — Each row shows the choices of a subject across different blocks (12 blocks). (PDF) [file pcbi.1006903.s007.pdf]

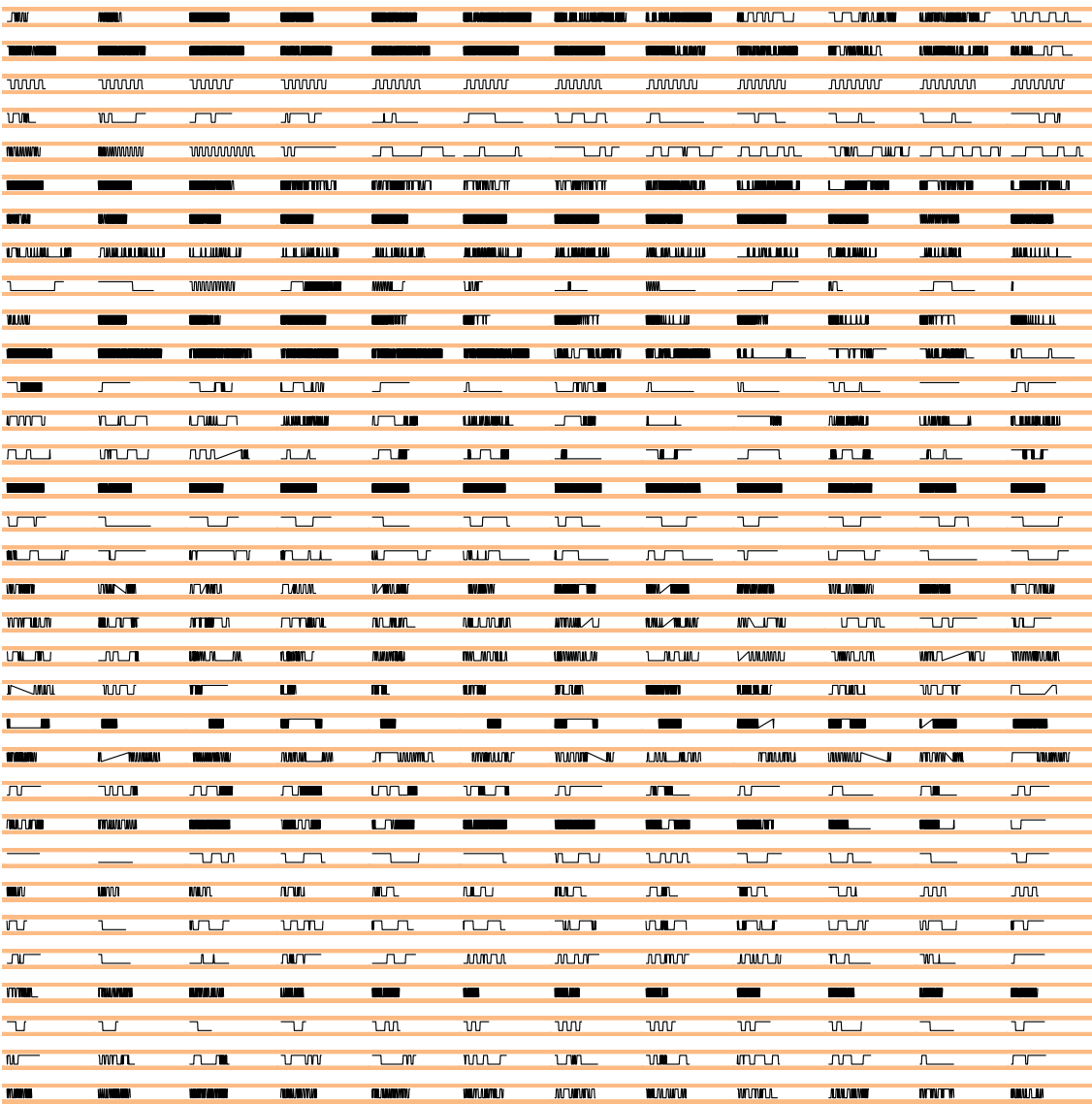

Supplement: S5 Fig — Each row shows the choices of a subject across different blocks (12 blocks). (PDF) [file pcbi.1006903.s008.pdf]

probability of selecting L

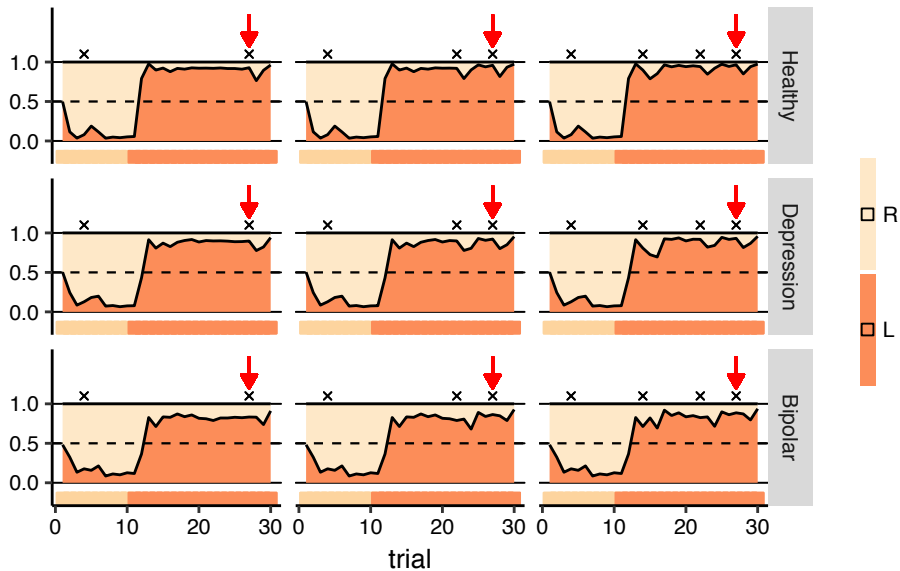

Supplement: S6 Fig — Each panel shows a simulation for 30 trials (horizontal axis), and the vertical axis shows the predictions for each group on each trial. The ribbon below each panel shows the action which was fed to the model on each trial. In the first 10 trials, the action that the model received was R and in the next 20 trials it was L. Rewards are shown by black crosses (x) on the graphs. See text for the interpretation of the graph. Note that the simulation conditions are same as those depicted in Figs 7 and 6. (PDF) [file pcbi.1006903.s009.pdf]

probability of selecting L

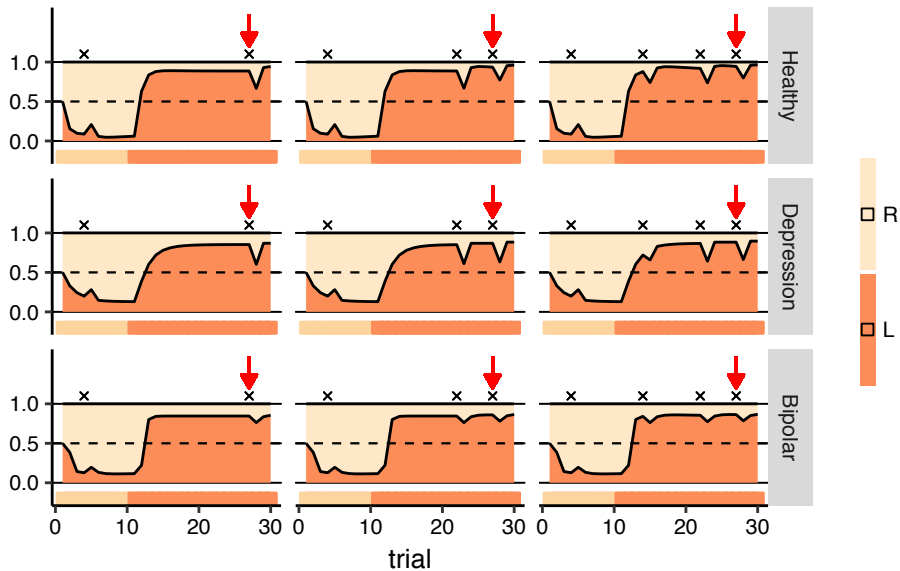

Supplement: S7 Fig — Each panel shows a simulation for 30 trials (horizontal axis), and the vertical axis shows the predictions for each group on each trial. The ribbon below each panel shows the action which was fed to the model on each trial. In the first 10 trials, the action that the model received was R and in the next 20 trials it was L. Rewards are shown by black crosses (x) on the graphs. See text for the interpretation of the graph. Note that the simulation conditions are the same as those depicted in Figs 7 and 6. (PDF) [file pcbi.1006903.s010.pdf]

probability of selecting L

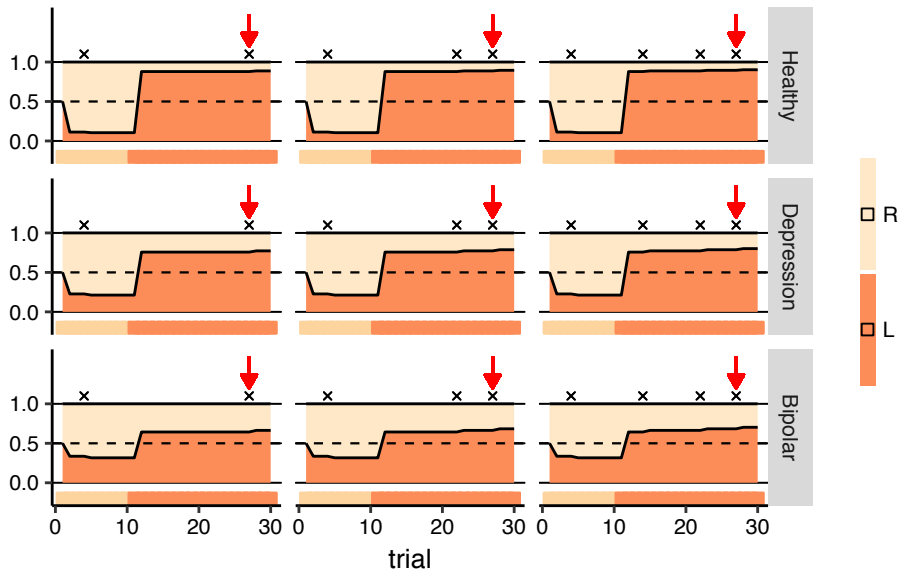

Supplement: S8 Fig — Each panel shows a simulation for 30 trials (horizontal axis), and the vertical axis shows the predictions for each group on each trial. The ribbon below each panel shows the action which was fed to the model on each trial. In the first 10 trials, the action that the model received was R and in the next 20 trials it was L. Rewards are shown by black crosses (x) on the graphs. See text for the interpretation of the graph. Note that the simulation conditions are the same as those depicted in Figs 7 and 6. (PDF) [file pcbi.1006903.s011.pdf]

probability of selecting L

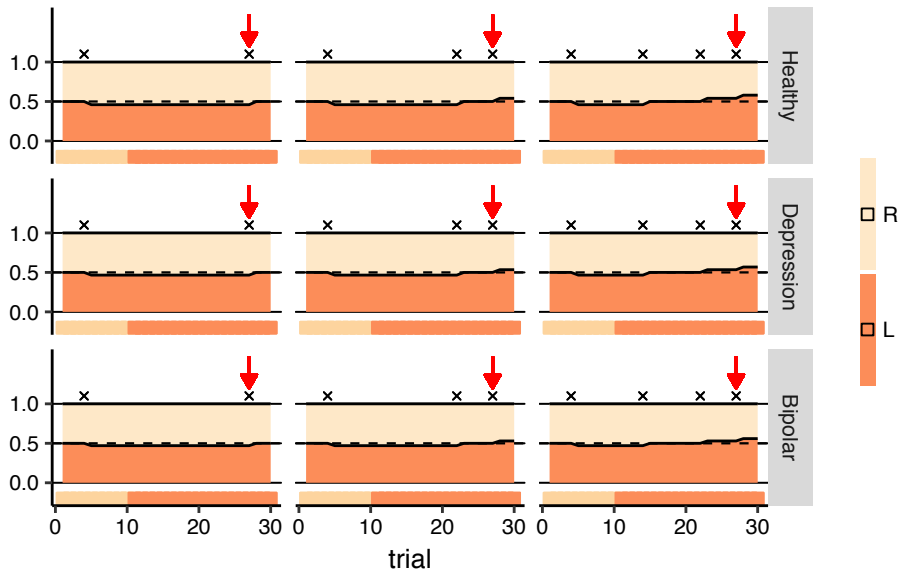

Supplement: S9 Fig — Each panel shows a simulation for 30 trials (horizontal axis), and the vertical axis shows the predictions for each group on each trial. The ribbon below each panel shows the action which was fed to the model on each trial. In the first 10 trials, the action that the model received was R and in the next 20 trials it was L. Rewards are shown by black crosses (x) on the graphs. See text for the interpretation of the graph. Note that the simulation conditions are the same as those depicted in Figs 7 and 6. (PDF) [file pcbi.1006903.s012.pdf]

probability of selecting L

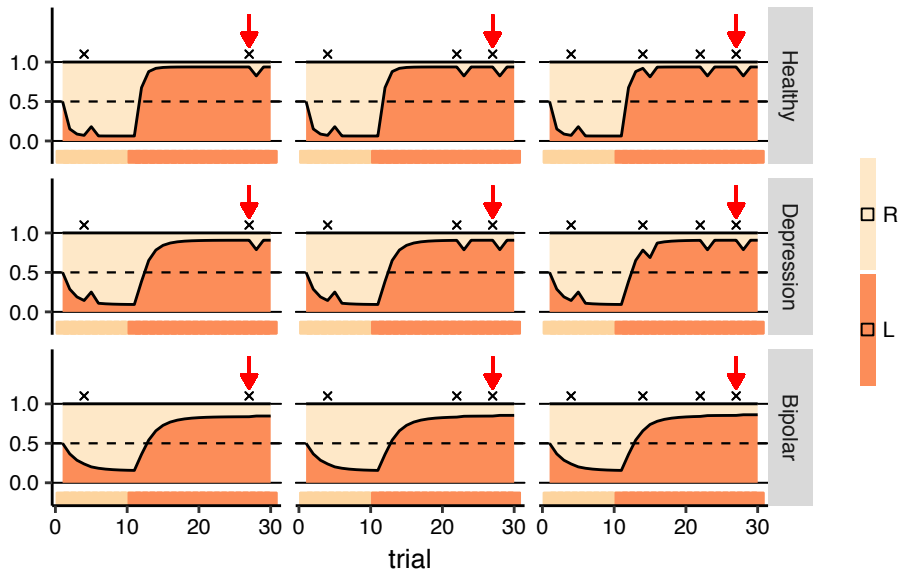

Supplement: S10 Fig — Each panel shows a simulation for 30 trials (horizontal axis), and the vertical axis shows the predictions for each group on each trial. The ribbon below each panel shows the action which was fed to the model on each trial. In the first 10 trials, the action that the model received was R and in the next 20 trials it was L. Rewards are shown by black crosses (x) on the graphs. See text for the interpretation of the graph. Note that the simulation conditions are the same as those depicted in Figs 7 and 6. (PDF) [file pcbi.1006903.s013.pdf]

probability of selecting L

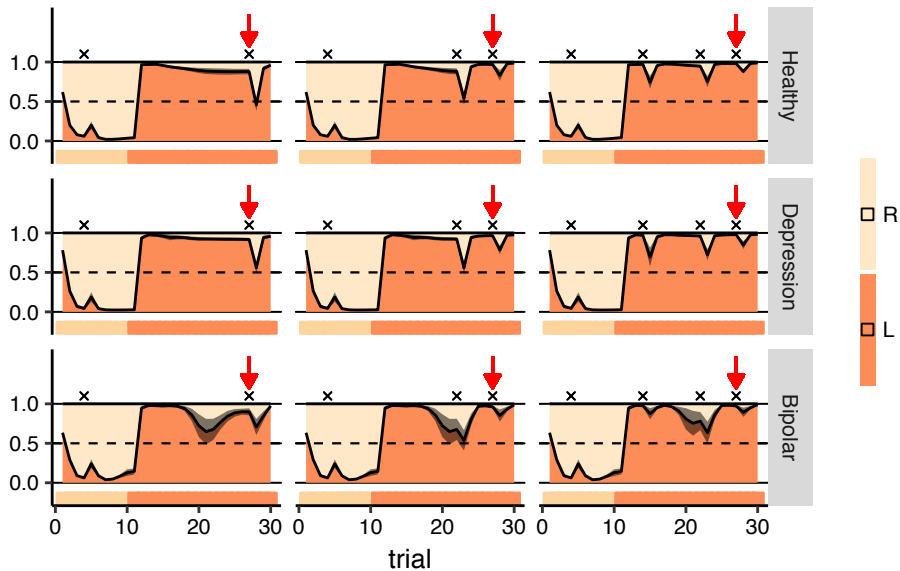

Supplement: S11 Fig — The simulation conditions are the same as those depicted in Figs 7 and 6. Here, 15 different initial networks were generated and optimised and the policies of the models on each trial were averaged. The grey ribbon around the policy shows the standard deviation of the policies. Each panel shows a simulation for 30 trials (horizontal axis), and the vertical axis shows the predictions of each model on each trial. The ribbon below each panel shows the action which was fed to the model on each trial. In the first 10 trials, the action that the model received was R and in the next 20 trials it was L. Rewards are shown by black crosses (x) on the graphs. See text for the interpretation of the graph. (PDF) [file pcbi.1006903.s014.pdf]

percentage of runs

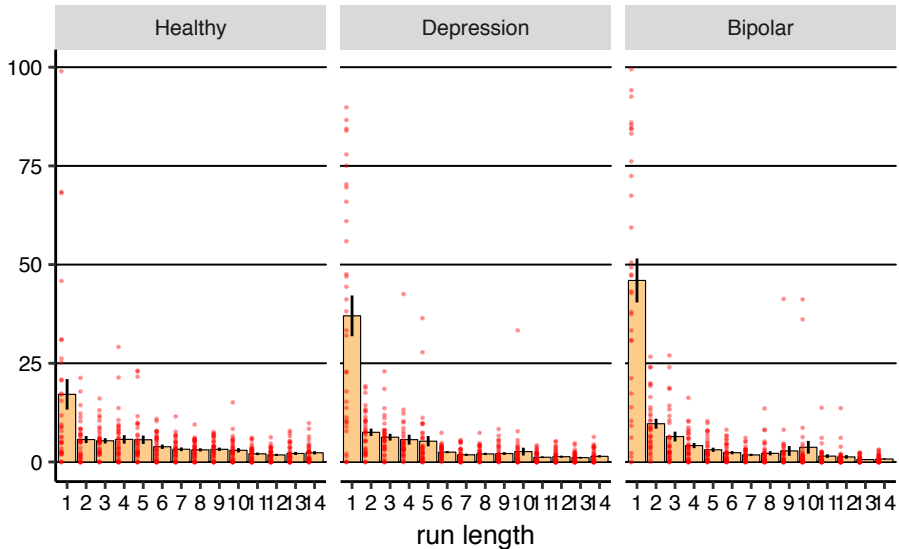

Supplement: S12 Fig — Percentage of each length of run of actions relative to the total number of runs in each subject (averaged over subjects). Red dots represent data for each subject, and error-bars represent 1SEM. (PDF) [file pcbi.1006903.s015.pdf]

stay probability

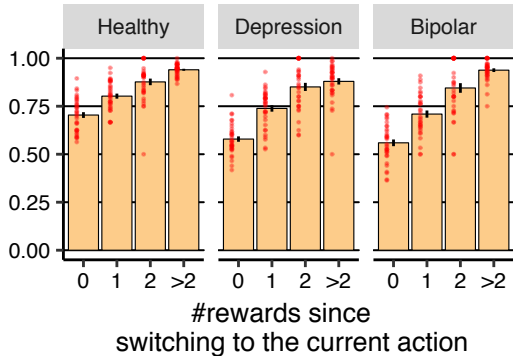

stay probability

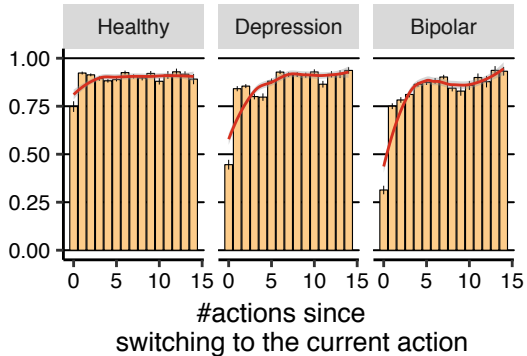

Supplement: S13 Fig — The graph is similar to Fig 8 but using data from rnn simulations (on-policy). (Left-panel) Probability of staying with an action after earning reward as a function of the number of actions taken since switching to the current action (averaged over subjects). Each red dot represents the data for each subject. (Right-panel) Probability of staying with an actions as a function of the number of actions taken since switching to the current action. The red line was obtained using Loess regression (Local Regression), which is a non-parametric regression approach. The grey area around the red line represents 95% confidence interval. Error-bars represent 1SEM. (PDF) [file pcbi.1006903.s016.pdf]

stay probability

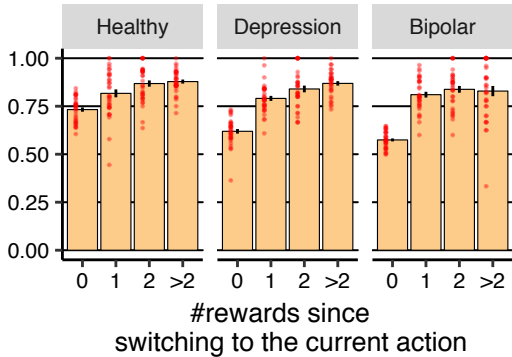

stay probability

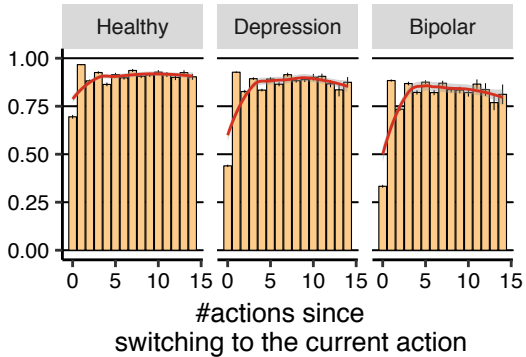

Supplement: S14 Fig — The graph is similar to Fig 8 but using data from lin simulations (on-policy). (Left-panel) Probability of staying with an action after earning reward as a function of the number of actions taken since switching to the current action (averaged over subjects). Each red dot represents the data for each subject. (Right-panel) Probability of staying with an actions as a function of the number of actions taken since switching to the current action. The red line was obtained using Loess regression (Local Regression), which is a non-parametric regression approach. The grey area around the red line represents 95% confidence interval. Error-bars represent 1SEM. (PDF) [file pcbi.1006903.s017.pdf]

stay probability

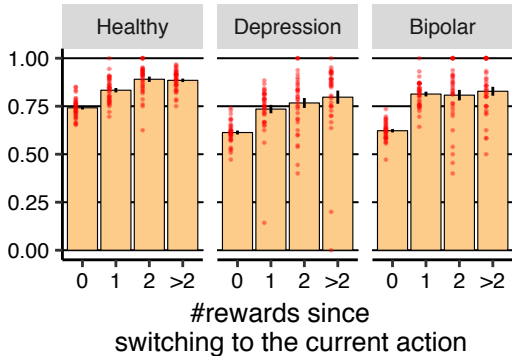

stay probability

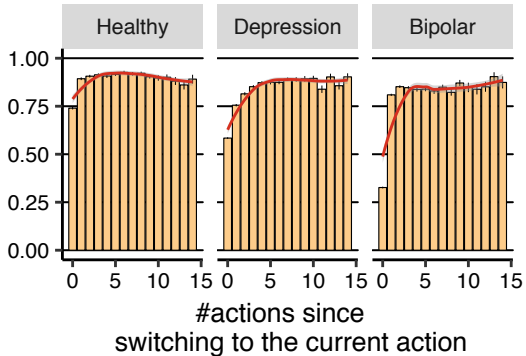

Supplement: S15 Fig — The graph is similar to Fig 8 but using data from gql simulations with d = 2 (on-policy). (Left-panel) Probability of staying with an action after earning reward as a function of the number of actions taken since switching to the current action (averaged over subjects). Each red dot represents the data for each subject. (Right-panel) Probability of staying with an actions as a function of the number of actions taken since switching to the current action. The red line was obtained using Loess regression (Local Regression), which is a non-parametric regression approach. The grey area around the red line represents 95% confidence interval. Error-bars represent 1SEM. (PDF) [file pcbi.1006903.s018.pdf]

length of current run

Healthy

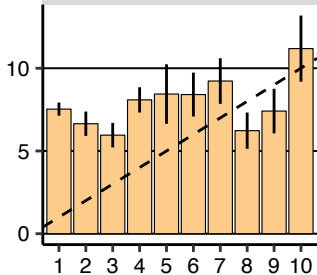

Depression

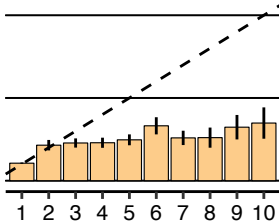

Bipolar

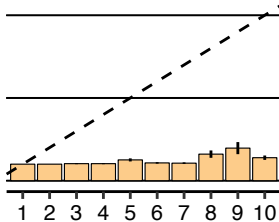

length of previous run

Supplement: S16 Fig — The graph is similar to Fig 9 but using data from gql simulations with d = 10 (on-policy). Median number of actions executed in a row before switching to another action (run of actions) in each subject as a function of the length of the previous run of actions (averaged over subjects). The dotted line shows the points at which the length of the previous and current runs are the same. Note that the median rather than the average was because we aimed to illustrate the most common ‘length of current run’, instead of average run length in each subject. Error-bars represent 1SEM. (PDF) [file pcbi.1006903.s019.pdf]

NLP

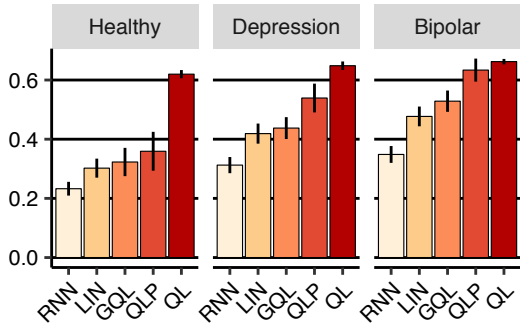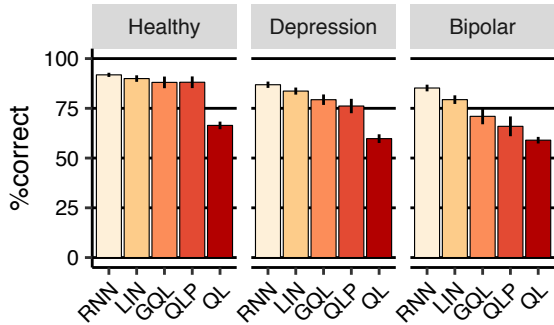

Supplement: S17 Fig — (Left-panel) nlp (negative log-probability) averaged across leave-one-out cross-validation folds. Lower values are better. (Right-panel) Percentage of actions predicted correctly averaged over cross-validation folds. Note that the difference between this figure and Fig 5 is that in Fig 5 hyper-parameters were obtained using in-sample estimations but here we used the data from two of the groups to obtain the optimal hyper-parameters (number of iterations/cells) for the other group. See text for more information. (PDF) [file pcbi.1006903.s020.pdf]
