# Supplementary material for: Models that learn how humans learn: The case of decision-making and its disorders
Source: PLoS Comput Biol. 2019 Jun 11;15(6):e1006903. doi: 10.1371/journal.pcbi.1006903 (PMC6588260; doi:10.1371/journal.pcbi.1006903)
Supplement: S1 Table — Number of subjects for each true- and predicted-label. The numbers inside parentheses are the percentage of subjects relative to the total number of subjects in each diagnostic group. (PDF) [file pcbi.1006903.s021.pdf]

Table S1. Prediction of diagnostic labels using LIN. Number of subjects for each true- and predicted-label. The numbers inside parentheses are the percentage of subjects relative to the total number of subjects in each diagnostic group.

		predicted labels		
		HEALTHY	DEPRESSION	BIPOLAR
true labels	HEALTHY	28 (82%)	2 (5%)	4 (11%)
	DEPRESSION	17 (50%)	5 (14%)	12 (35%)
	BIPOLAR	12 (36%)	7 (21%)	14 (42%)
