# Supplementary material for: Models that learn how humans learn: The case of decision-making and its disorders
Source: PLoS Comput Biol. 2019 Jun 11;15(6):e1006903. doi: 10.1371/journal.pcbi.1006903 (PMC6588260; doi:10.1371/journal.pcbi.1006903)
Supplement: S2 Table — Number of subjects for each true- and predicted-label. The numbers inside parentheses are the percentage of subjects relative to the total number of subjects in each diagnostic group. (PDF) [file pcbi.1006903.s022.pdf]

**Table S2. Prediction of diagnostic labels using GQL ($d = 2$).** Number of subjects for each true- and predicted-label. The numbers inside parentheses are the percentage of subjects relative to the total number of subjects in each diagnostic group.

| | | predicted labels | | |
|---|---|---|---|---|
| | | HEALTHY | DEPRESSION | BIPOLAR |
| true labels | HEALTHY | 29 (85%) | 2 (5%) | 3 (8%) |
| | DEPRESSION | 16 (47%) | 7 (20%) | 11 (32%) |
| | BIPOLAR | 12 (36%) | 6 (18%) | 15 (45%) |
