# Supplementary material for: Models that learn how humans learn: The case of decision-making and its disorders
Source: PLoS Comput Biol. 2019 Jun 11;15(6):e1006903. doi: 10.1371/journal.pcbi.1006903 (PMC6588260; doi:10.1371/journal.pcbi.1006903)
Supplement: S3 Table — (PDF) [file pcbi.1006903.s023.pdf]

Table S3. Estimated parameters for QL model.

	α	β	$\alpha\beta$
HEALTHY	0.0002	616.559	0.162
DEPRESSION	0.00001	11760.3	0.14
BIPOLAR	0.00002	4025.94	0.12
