# Supplementary material for: Models that learn how humans learn: The case of decision-making and its disorders
Source: PLoS Comput Biol. 2019 Jun 11;15(6):e1006903. doi: 10.1371/journal.pcbi.1006903 (PMC6588260; doi:10.1371/journal.pcbi.1006903)
Supplement: S4 Table — (PDF) [file pcbi.1006903.s024.pdf]

Table S4. Estimated parameters for QLP model.

	α	β	κ	$\alpha\beta$
HEALTHY	0.0008	98.8895	2.0634	0.079
DEPRESSION	0.00003	2479.31	1.223	0.09
BIPOLAR	0.00008	1113.97	0.680	0.09
