# Supplementary material for: Models that learn how humans learn: The case of decision-making and its disorders
Source: PLoS Comput Biol. 2019 Jun 11;15(6):e1006903. doi: 10.1371/journal.pcbi.1006903 (PMC6588260; doi:10.1371/journal.pcbi.1006903)
Supplement: S5 Table — (PDF) [file pcbi.1006903.s025.pdf]

Table S5. Estimated parameters for GQL model with $d = 2$.

	**Φ**	**Ψ**	**B**	**K**	**C**
HEALTHY	[0.145 0.815]	[0.635 0.389]	[4.258 -1.002]	[3.268 -0.974]	[[-14.256 4.243]<br>[17.998 -6.335]]
DEPRESSION	[0.003 0.999]	[0.399 0.3199]	[8.691 -0.315]	[1.709 0.077]	[[14.918 6.112]<br>[19.599 -7.292]]
BIPOLAR	[0.147 0.654]	[0.897 0.999]	[4.363 -1.1453]	[14.447 -12.501]	[[0.174 -15.199]<br>[-2.936 15.164]]
