# Supplementary material for: Models that learn how humans learn: The case of decision-making and its disorders
Source: PLoS Comput Biol. 2019 Jun 11;15(6):e1006903. doi: 10.1371/journal.pcbi.1006903 (PMC6588260; doi:10.1371/journal.pcbi.1006903)
Supplement: S6 Table — (PDF) [file pcbi.1006903.s026.pdf]

Table S6. Negative log-likelihood for each model optimized over all the subjects in each group.

	RNN	GQL	QLP	QL
HEALTHY	9421.6660	12939.40	14557.44	27616.79
DEPRESSION	13158.1074	19735.61	23378.65	29862.19
BIPOLAR	12891.3496	19363.08	24859.15	26843.88
