# Supplementary material for: Models that learn how humans learn: The case of decision-making and its disorders
Source: PLoS Comput Biol. 2019 Jun 11;15(6):e1006903. doi: 10.1371/journal.pcbi.1006903 (PMC6588260; doi:10.1371/journal.pcbi.1006903)
Supplement: S7 Table — For rnn a single model was fitted to the whole group using ML estimation. For baseline methods (gql, qlp, and ql), a separate model was fitted to each subject, and the reported number is the sum of negative log-likelihoods over the whole group. (PDF) [file pcbi.1006903.s027.pdf]

Table S7. Negative log-likelihood for each model. For RNN a single model was fitted to the whole group using ML estimation. For baseline methods (GQL, QLP, and QL), a separate model was fitted to each subject, and the reported number is the sum of negative log-likelihoods over the whole group.

	RNN	GQL	QLP	QL
HEALTHY	9421.6660	9482.846	11529.58	26080.13
DEPRESSION	13158.1074	14668.763	17837.02	28448.94
BIPOLAR	12891.3496	14157.206	16912.37	25874.56
