# Supplementary material for: Models that learn how humans learn: The case of decision-making and its disorders
Source: PLoS Comput Biol. 2019 Jun 11;15(6):e1006903. doi: 10.1371/journal.pcbi.1006903 (PMC6588260; doi:10.1371/journal.pcbi.1006903)
Supplement: S8 Table — (PDF) [file pcbi.1006903.s028.pdf]

Table S8. Mean and standard deviation of negative log-likelihood for RNN over 15 different initialisations of the model and optimised over all the subjects in each group.

	mean (standard deviation)
HEALTHY	9450.937 (81.512)
DEPRESSION	13268.186 (135.499)
BIPOLAR	12885.629 (134.552)
