# Supplementary material for: Models that learn how humans learn: The case of decision-making and its disorders
Source: PLoS Comput Biol. 2019 Jun 11;15(6):e1006903. doi: 10.1371/journal.pcbi.1006903 (PMC6588260; doi:10.1371/journal.pcbi.1006903)
Supplement: S9 Table — (PDF) [file pcbi.1006903.s029.pdf]

Table S9. Average number of trials in each pair of reward probabilities in each group.

Group	Number of trials	Pair of probabilities
Bipolar	108.06	[0.08, 0.05]
Bipolar	103.90	[0.05, 0.125]
Bipolar	96.42	[0.25, 0.05]
Depression	119.58	[0.08, 0.05]
Depression	117.13	[0.05, 0.125]
Depression	108.02	[0.25, 0.05]
Healthy	113.61	[0.08, 0.05]
Healthy	112.22	[0.05, 0.125]
Healthy	102.53	[0.25, 0.05]
