# Supplementary material for: Models that learn how humans learn: The case of decision-making and its disorders
Source: PLoS Comput Biol. 2019 Jun 11;15(6):e1006903. doi: 10.1371/journal.pcbi.1006903 (PMC6588260; doi:10.1371/journal.pcbi.1006903)
Supplement: S10 Table — (PDF) [file pcbi.1006903.s030.pdf]

Table S10. Mean of NLP derived using in-sample hyper-parameter estimation (in-sample) and using the data of other groups (other-groups) .

Group	in-sample	other-groups
HEALTHY	0.2323	0.2326
DEPRESSION	0.3114	0.3124
BIPOLAR	0.3480	0.3484
